# Evolutionary History of Atmospheric CO_2_ during the Late Cenozoic from Fossilized *Metasequoia* Needles

**DOI:** 10.1371/journal.pone.0130941

**Published:** 2015-07-08

**Authors:** Yuqing Wang, Arata Momohara, Li Wang, Julie Lebreton-Anberrée, Zhekun Zhou

**Affiliations:** 1 Key Laboratory of Tropical Forest Ecology, Xishuangbanna Tropical Botanical Garden, Chinese Academy of Sciences, Mengla 666303, China; 2 University of Chinese Academy of Sciences, Beijing 100049, China; 3 Key Laboratory of Plant Diversity and Biogeography of East Asia, Kunming Institute of Botany, CAS, Kunming 650204, China; 4 Graduate School of Horticulture, Chiba University, 648 Matsudo, Chiba 271–8510, Japan; 5 Central Laboratory, Xishuangbanna Tropical Botanical Garden, Chinese Academy of Sciences, Mengla 666303, China; Institute of Botany, CHINA

## Abstract

The change in ancient atmospheric CO_2_ concentrations provides important clues for understanding the relationship between the atmospheric CO_2_ concentration and global temperature. However, the lack of CO_2_ evolution curves estimated from a single terrestrial proxy prevents the understanding of climatic and environmental impacts due to variations in data. Thus, based on the stomatal index of fossilized *Metasequoia* needles, we reconstructed a history of atmospheric CO_2_ concentrations from middle Miocene to late Early Pleistocene when the climate changed dramatically. According to this research, atmospheric CO_2_ concentration was stabile around 330–350 ppmv in the middle and late Miocene, then it decreased to 278–284 ppmv during the Late Pliocene and to 277–279 ppmv during the Early Pleistocene, which was almost the same range as in preindustrial time. According to former research, this is a time when global temperature decreased sharply. Our results also indicated that from middle Miocene to Pleistocene, global CO_2_ level decreased by more than 50 ppmv, which may suggest that CO_2_ decrease and temperature decrease are coupled.

## Introduction

Carbon dioxide (CO_2_) is an important greenhouse gas that influences the surface temperature of the Earth [1]. The 5^th^ report of IPCC concluded [2] that the present positive radiative forcing is unequivocally caused by anthropogenic increases in atmospheric CO_2_ concentration and that it influences the climate [3,4]. Estimating the impact of high CO_2_ concentration on global environmental systems is the first step to propose solutions for the present global climate change. This impact can be unraveled by a better understanding of the relationship between the paleo-atmospheric CO_2_ concentration (paleo-[CO_2_]_atm_) and ancient climate change.

A lot of research has involved the estimation of paleo-[CO_2_]_atm_ to understand the correlation between CO_2_ and global warming. To obtain the paleo-[CO_2_]_atm_ values three major approaches have been used: (1) geochemical modeling (GCS) [5–7], (2) composition measurements of air trapped in ice cores [8], and (3) various proxies (reviewed in [9]). Geochemical modeling (GCS) can reconstruct paleo-[CO_2_]_atm_, but for long geological time scales its resolution cannot be fine enough to show the details of paleo-[CO_2_]_atm_ fluctuation [10]. Ice core analysis is the most reliable method to measure paleo-[CO_2_]_atm_ directly, but is only applicable after 0.8 Ma [8]. Several CO_2_ proxies have been used to estimate paleo-[CO_2_]_atm_, such as the carbon isotope composition of phytoplankton, the boron (B) isotope composition of fossil foraminifera, the carbon isotope composition of carbonates in paleosol, and the stomatal parameters of fossil leaves [11]. High resolution records for CO_2_ can be obtained from marine sediments with the two former proxies, but these do not directly show the paleo-[CO_2_]_atm_. The latter two proxies are terrestrial-based proxies that reflect paleo-[CO_2_]_atm_ directly, although they rarely provide continuous paleo-[CO_2_]_atm_ records for a long geological time. Therefore, while there is a consensus on the general tendency of the Cenozoic paleo-[CO_2_]_atm_ changes, the estimated paleo-[CO_2_]_atm_ values vary greatly [9]. To understand the paleoclimatic system, it is important to reduce uncertainties in the relationships between paleo-[CO_2_]_atm_ and past climate [12].

Stomatal parameters (SI (stomatal index) and SD (stomatal density)) are reliable proxies to estimate paleo-[CO_2_]_atm_. In particular, SI can provide a robust indicator of terrestrial paleo-[CO_2_]_atm_ as it is independent of other environmental parameters, such as soil moisture supply, atmospheric humidity and temperature [13]. Many studies have already used the SI of different taxa to estimate paleo-[CO_2_]_atm_, such as *Metasequoia* Miki *ex* Hu *et* Cheng [12,14], *Ginkgo* Linn. [15,16], *Quercus* Linn. [17,18], *Laurus* Linn. [17,19,20], *Platanus* Linn. [17,21], and *Typha* Linn. [22]. As the relationship between the SI and paleo-[CO_2_]_atm_ is species-specific even within a single family [23] and the response sensitivities to CO_2_ change are different in various taxa [24], it is necessary to select a single modern taxon that has survived for an extended period to reconstruct atmospheric CO_2_ over a long geological time.

*Metasequoia* has exhibited an evolutionary stasis since its appearance in the Late Cretaceous [25], and fossilized *Metasequoia* can be considered to be conspecific with modern *Metasequoia* based on the morphology, biochemistry and inferred physiology [26]. Therefore, the paleo-[CO_2_]_atm_ changes over a long geological time can be determined from a correlation between the SI of *Metasequoia* needles and the paleo-[CO_2_]_atm_ concentration [14].

In this study, we use *Metasequoia* needles from seven localities in China and Japan to reconstruct continuous terrestrial paleo-[CO_2_]_atm_ changes from the middle Miocene to Pleistocene. Based on the reconstructed paleo-[CO_2_]_atm_ curve, we discuss the interaction between paleo-[CO_2_]_atm_ evolution and global environment change since the middle Miocene.

## Materials and Methods

### Materials

**Fig 1:**
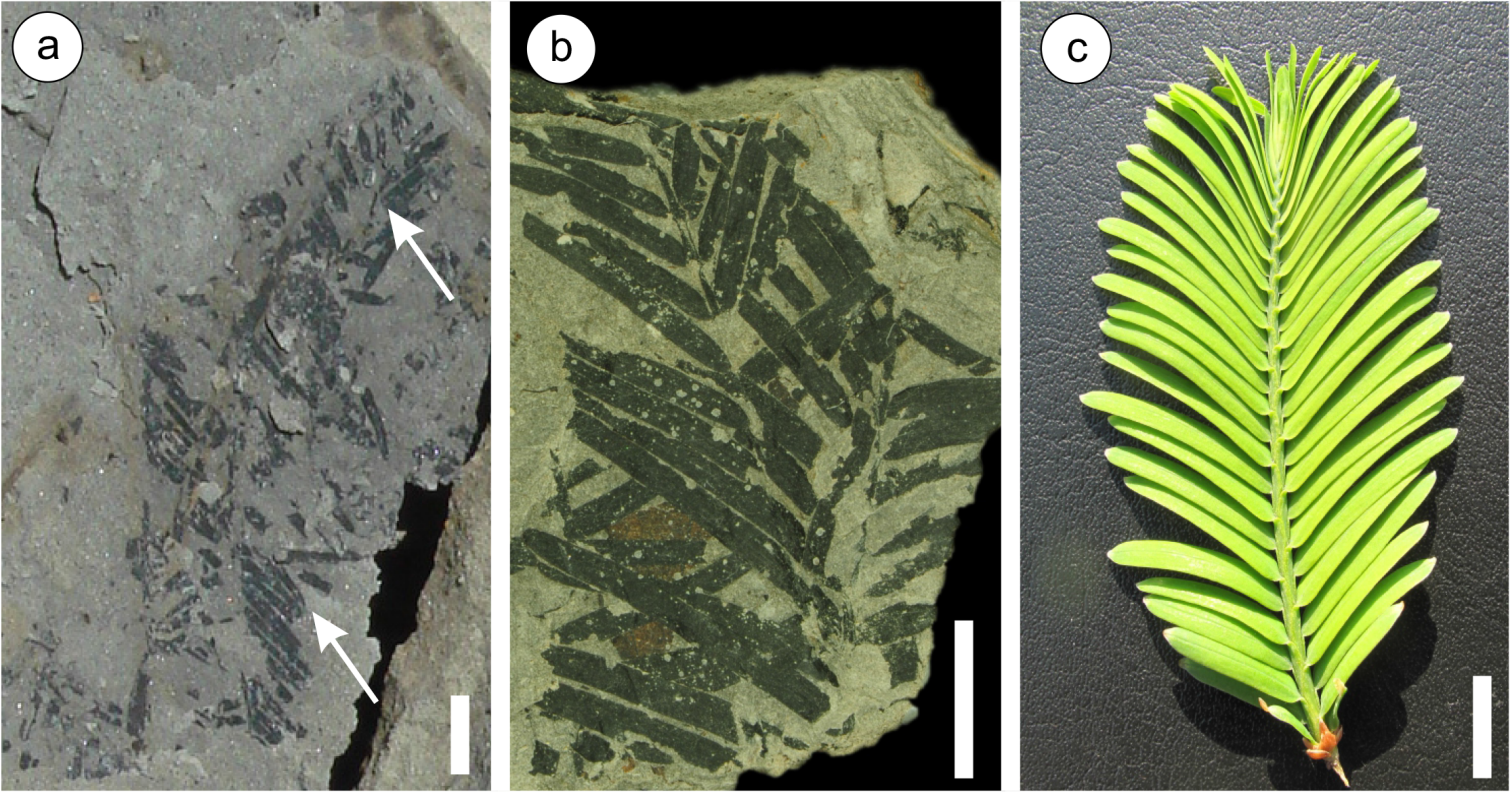
Fossilized *Metasequoia* from Tokamachi and Kumagaya sites. Fossilized *Metasequoia* branchlet and needles from Tokamachi (a) and Kumagaya (b) as examples to show the megafossils of *Metasequoia* used in this research, compare with a modern *Metasequoia* branchlet (c). White arrows in (a) indicate the branchlet.

**Fig 2:**
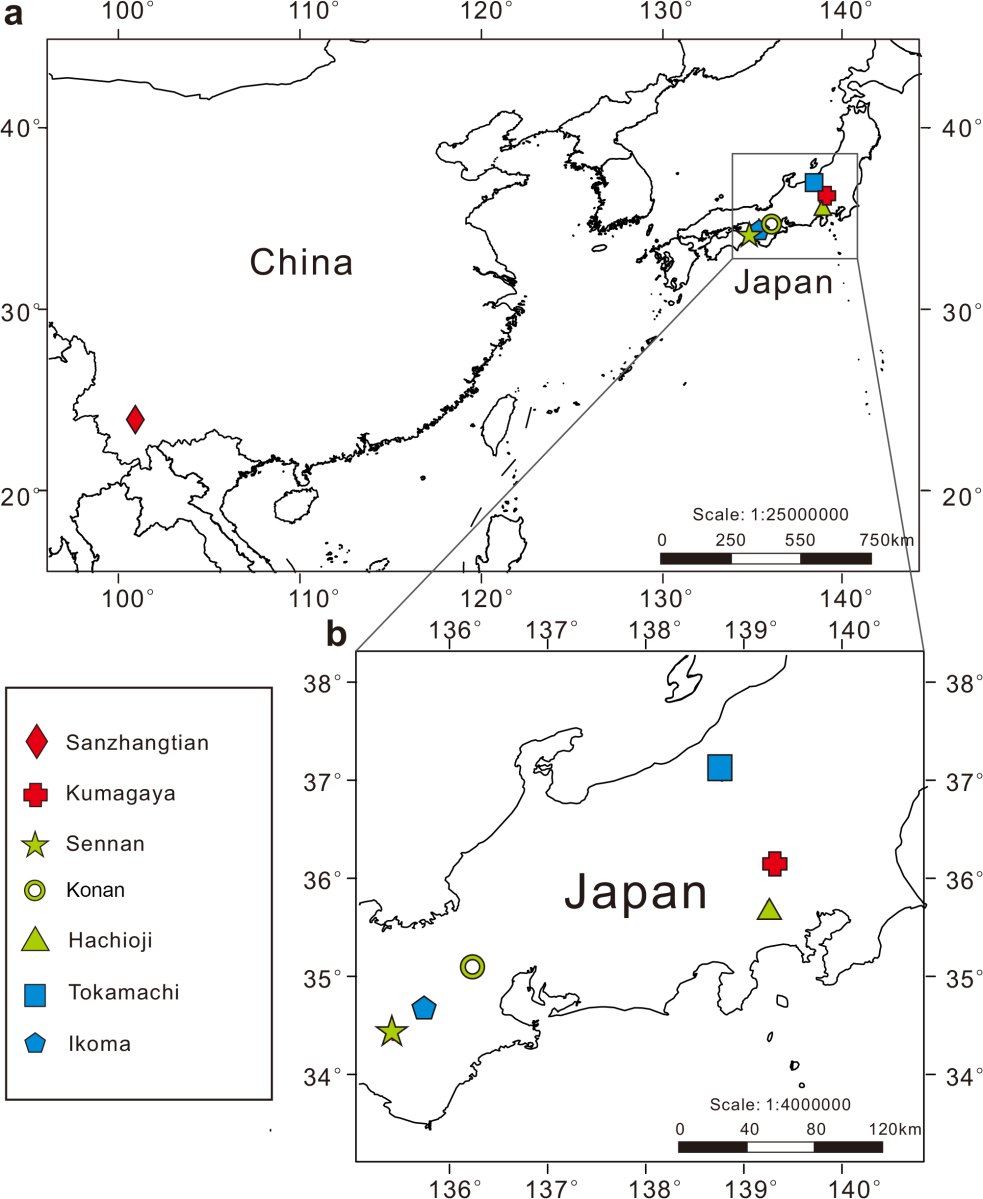
Localities where fossilized *Metasequoia* were obtained. Locality map (a) showing the seven fossil sites in China and Japan. Enlarged map (b) illustrating the central area of Japan showing the position of the six localities in Japan: Kumagaya, Sennan, Konan, Hachioji, Tokamachi and Ikoma. Different colors identify the different ages of the localities (Red: Miocene; Green: Pliocene; Blue: Pleistocene).

The fossilized needles of *Metasequoia* (Fig 1) were collected from one locality in SW China (Sanzhangtian) and six localities in central Japan (Kumagaya, Sennan, Hachioji, Konan, Tokamachi, and Ikoma sites) (Fig 2, Table 1). We confirm that our field study did not involve endangered or protected species and none of the localities which provided samples for this study are in protected areas. The Sanzhangtian locality belongs to the National land of the People's Republic of China, and the Land and Resources Bureau of Zhenyuan County gave permission to collect fossils from this locality. The Japanese sites: Kumagaya, Hachioji, Konan, and Tokamachi are on valley floors which are public space, so no permission was required to conduct sampling. The Sennan and Ikoma sites belong to private owners, who gave permission for sampling.

**Table 1:** *Metasequoia* samples used for reconstructing paleo-CO_2_ including fossil sites, ages, latitude, and longitude.

Fossil site	Locality	Latitude/ Longitude	Geologic setting	Epoch	Absolute age	Dating method	Voucher specimens #	Remark	Reference
Sanzhangtian	Yunnan, China	24°06′ N, 101°13′ E	Dajie Formation	middle Miocene	10–16 Ma	Stratigraphic study	SZT077,SZT156,SZT115,SZT127,SZT123,SZT126		[27–29]
Kumagaya	Saitama Prefecture, Japan	36°08′ N, 139°18′ E	Yagii Formation in the Matsuyama Group	early late Miocene	9–10 Ma	Zircon fission track dating	YJ003,YJ005	Includes marine bed	[30]
Sennan	Osaka Prefecture, Japan	34°24' N, 135°28' E	Lower than the Habutaki I Tephra, Osaka Group	Late Pliocene	2.8–3.0 Ma	Magnetostratigraphy and calcareous nanoplankton stratigraphy	FT001	Included in sediments in fluvial backmarsh	[31–33]
Hachioji	Tokyo, Japan.	35° 40′ N, 139°18′ E	Kasumi Formation (below the Gauss and Matuyama Chron boundary)	Late Pliocene	2.6–2.7 Ma	Magnetostratigraphy and calcareous nanoplankton stratigraphy	BQC001	Includes marine bed	[34,35]
Konan	Shiga Prefecture, Japan	34°59′ N, 136°6′ E	Horizon correlated with the Kamide I tephra bed in Kobiwako Group (just below the Gauss and Matuyama Chron boundary)	Late Pliocene	2.6 Ma	Magnetostratigraphy and calcareous nanoplankton stratigraphy	SG001,SG002	Included in sediments in fluvial backmarsh	[36]
Tokamachi	Niigata Prefecture, Japan.	37°07'N, 138°48'E	Middle part of the Uonuma Group lower part of Olduvai paleomagnetic chron	middle Early Pleistocene	1.85 Ma	Magnetostratigraphy and calcareous nanoplankton stratigraphy	156u01	Includes marine bed	[32,37,38]
Ikoma	Nara Prefecture, Japan	34°44'N, 135°43'E	Peat layer just below the Ma 2 Marine Clay bed (MIS 25) in the Osaka Group	latest Early Pleistocene	0.95 Ma	Magnetostratigraphy and calcareous nanoplankton stratigraphy	NR001	Includes marine bed	[39,40]

*Metasequoia* fossils had previously been reported from all the fossil localities. Their ages were estimated based on stratigraphic studies (Sanzhangtian site), zircon fission-track methods (Kumagaya site), and regional stratigraphic correlation using magnetostratigraphy and calcareous nanoplankton stratigraphy (Sennan, Hachioji, Konan, Tokamachi, and Ikoma sites) (Table 1). For the samples, two were from the Miocene (Sanzhangtian and Kumagaya), three from the upper Pliocene (Sennan, Hachioji, and Konan), and two from the lower Pleistocene (Tokamachi and Ikoma) (Table 1). At least six different needles from different branchlets were used in the studies from each site, and the exact amount depends on the total amount of materials at each fossil site (Table 2).

**Table 2:** Sample size of localities.

Fossil site	Epoch	Sample size (no.)
Sanzhangtian	middle Miocene	25
Kumagaya	early late Miocene	11
Sennan	Late Pliocene	6
Hachioji	Late Pliocene	8
Konan	Late Pliocene	7
Tokamachi	middle Early Pleistocene	17
Ikoma	latest Early Pleistocene	7

Voucher specimens from the Sanzhangtian, Kumagaya, Hachioji, Konan, and Tokamachi sites are housed in the Herbarium of Kunming Institute of Botany (KUN), Chinese Academy of Science. Specimens from the Sennan and Ikoma sites are housed in the Graduate School of Horticulture, Chiba University, Japan.

## Methods

### Pretreatment of the fossilized needles

To remove the inorganic compounds adhering to the fossilized needles, the material was first immersed in 10%–25% Hydrochloric Acid (HCl) for two hours, then in 40% Hydrofluoric Acid (HF) for 12 hours, and in 10%–25% HCl for at least one hour. The needles were then rinsed with distilled water and divided into three parts, and the central piece (when available) used to obtain the cuticle.

### Cleaned cuticular membrane maceration

For the material from the Sanzhangtian, Kumagaya, and Konan sites, we followed the methods of Kerp [41] to isolate the lower cuticle of the fossilized needles. (1) The specimens were first macerated with 70% Nitric acid (HNO_3_) for between a few minutes to an hour until they turned yellowish-brown. (2) Once it had been rinsed with distilled water several times, (3) the upper and the lower epidermis were separated using a needle. (4) Then, the epidermis was treated with a 3%–5% Sodium Hypochlorite (NaClO) solution for around 10 minutes to remove the remnants of the mesophyll, vascular bundle, hypodermal layer, and epidermal cell walls. (5) According to the state of the material, 5%-10% Aqueous Ammonia (NH_3_·H_2_O) or 30% Hydrogen Peroxide (H_2_O_2_) can be used instead of the 3%–5% NaClO. (6) Finally glycerol was used to mount the separated cuticles for observation.

### Cuticle observation and photography

The separated cuticles of the material from the Sanzhangtian, Kumagaya, and Konan sites were observed using a transmitted light microscope (Zeiss Axio Imager A2) and photographed with a digital camera (Zeiss AxioCam MRc). For the materials from the Sennan, Hachioji, Tokamachi, and Ikoma sites, pretreated fossilized leaves were mounted with water on slides and the lower sides of the needles were directly scanned by a confocal laser scanning microscope (Zeiss LSM710, Imager. Z2, Ar Lasser 488nm). Each field-of-view was larger than 0.03mm^2^ [42]. Photoshop (version CS6, Adobe Systems; Mountain View, CA) was used to merge 6–12 serial images that were taken of the same area but at different focal levels.

### Measurement of SI and paleo-[CO_2_]_atm_ concentration

Image J (1.43μ, Wayne Rasband, http://rsb.info.nih.gov/ij/) was used to calculate the number of epidermal cells and stomatal complexes (stomatal pore + guard cells). Then, the SI was calculated using Eq 1 [43].

$$SI=\frac{stomatal\;complexes\;number}{epidermal\;cell\;number+stomatal\;complexes\;number}\times 100\%$$ Equation 1

The SI data were used to estimate the paleo-[CO_2_]_atm_ from the middle Miocene to Pleistocene by using the species-specific, nonlinear negative correlation between atmospheric CO_2_ partial pressure and SI (Eq 2) based on Royer et al. [14].

$$Paleo-{\left[CO_{2}\right]}_{atm}=\frac{SI-6.672}{0.003883\times SI-0.02897}$$ Equation 2

The significant differences between the mean variance of the SI from different ages were statistically tested using the two tailed one-way ANOVA with the “LSD” option in IBM SPSS Statistics (Version 20.0).

## Results

**Fig 3:**
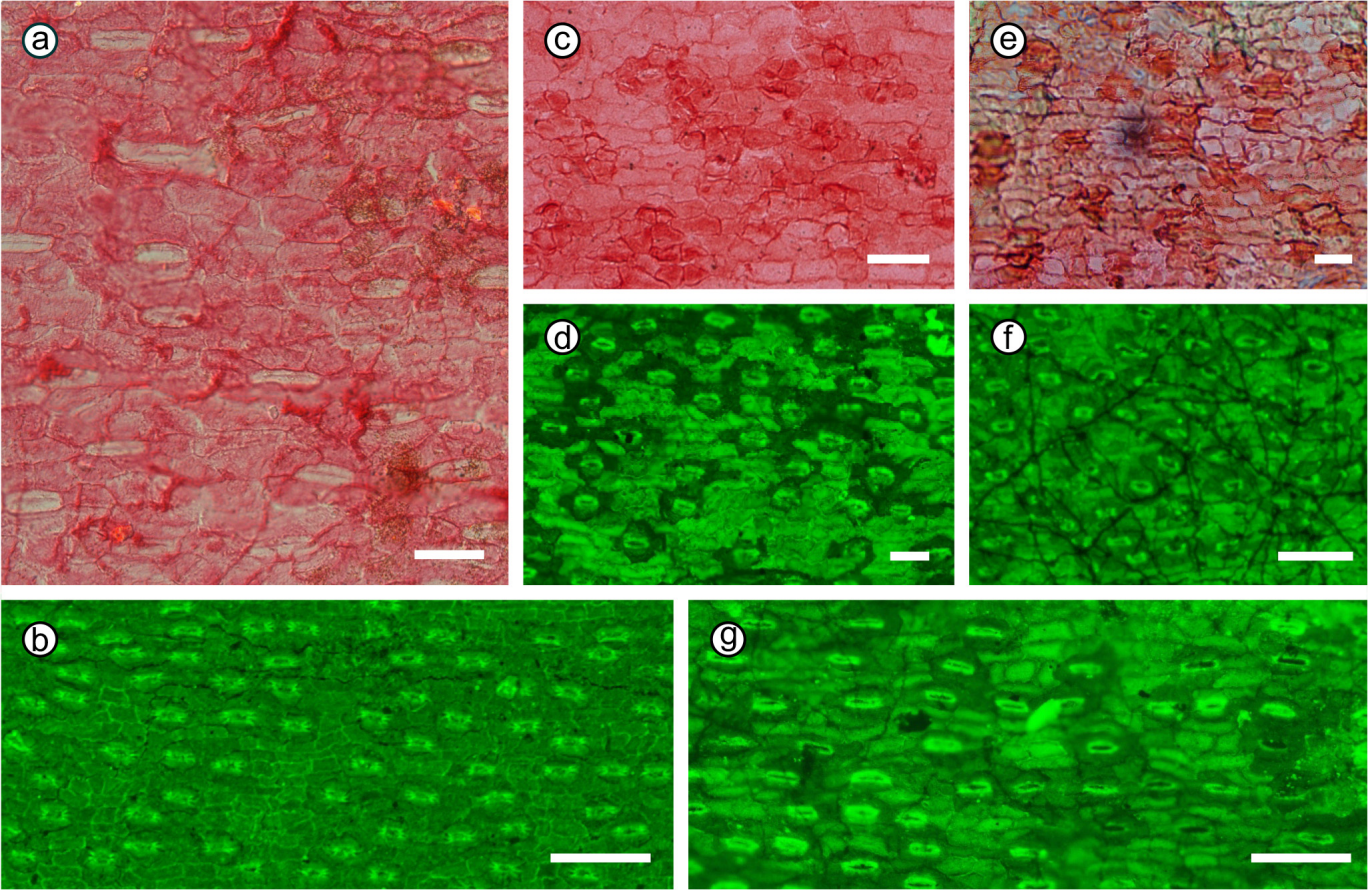
Lower cuticles of the *Metasequoia* needle samples from different localities. Lower cuticles of *Metasequoia* needles from a: Sanzhangtian; b: Ikoma; c: Kumagaya; d: Tokamachi; e: Konan; f: Sennan; and g: Hachioji. (Scale Bar = 100μm)

Fossilized *Metasequoia* needles from the early late Miocene Kumagaya site had the lowest SI value (SI = 9.80 ± 0.65) and those from the middle Miocene Sanzhangtian site had the second lowest (SI mean = 10.43 ± 0.99). Their calculated paleo-[CO_2_]_atm_ values were 351 ± 24.8 ppmv and 334 ± 24.8 ppmv, respectively (Fig 3, Table 3, for more details see S1 Table).

**Table 3:** Fossilized *Metasequoia* stomatal index and paleo-[CO_2]atm_ concentration estimates during Cenozoic.

Fossil site	Epoch	SI (%)			paleo-[CO_2_]_atm_ (ppmv)		
		Mean ± sd	Max	Min	Mean ± sd	Max	Min
Sanzhangtian	middle Miocene	10.4±0.99	12.5	9.09	334±24.9	382	298
Kumagaya	early late Miocene	9.80±0.65	11.0	8.97	351±24.8	392	317
Sennan	Late Pliocene	17.1±2.30	19.8	14.8	280±5.16	285	274
Hachioji	Late Pliocene	17.2±1.65	19.4	14.8	279±3.74	285	275
Konan	Late Pliocene	15.2±1.70	18.4	13.7	285±5.15	290	276
Tokamachi	middle Early Pleistocene	17.1±2.52	21.7	13.3	280±6.23	293	272
Ikoma	latest Early Pleistocene	17.9±1.90	20.2	15.5	278±3.86	282	273

The SI of the Pliocene and Pleistocene samples were higher (SI mean = 15.2–17.9) than the SI of the Miocene samples. The SI of the samples from the middle Early Pleistocene Ikoma site had the highest SI value (SI = 17.9 ± 1.9) and give out the lowest CO_2_ level of 278 ± 3.86 ppmv. SI of the fossilized leaves from the Sennan, Hachioji, Konan, and Tokamachi sites were 17.1, 17.2, 15.2, and 17.1, respectively. The reconstructed paleo-[CO_2_]_atm_ from the Pliocene and Pleistocene samples in the Sennan, Hachioji, Konan, Tokamachi, and Ikoma sites were 280 ± 5.16, 279 ± 3.74, 285 ± 5.15, 280 ± 6.23, and 278 ± 3.86 ppmv, respectively (Fig 3, Table 3, for more details see S1 Table).

The significant differences between the mean variance of the stomatal index from different fossil localities were statistically tested (F = 54.016, p<0.001) by one-way ANOVA with the “LSD” option in SPSS Statistics (Version 20.0). The result showed there was no significant difference between the SI data from Sanzhangtian locality (middle Miocene) and Kumagaya locality (late Miocene), but the SI data of these two localities were significantly different from the SI data from late Pliocene and Pleistocene localities. SI data of Konan locality (Late Pliocene) was significantly different from all other localities, but no significant difference has been detected among Sennan (Late Pliocene), Hachioji (Late Pliocene), Tokamachi (middle Early Pleistocene) and Ikoma localities (latest Early Pleistocene) (Table 4).

**Table 4:** Mean difference of the least significant different (LSD) on stomatal index of fossil localities.

Locality	Sanzhangtian	Kumagaya	Sennan	Hachioji	Konan	Tokamachi
**Kumagaya**	0.63					
**Sennan**	**-6.70** ***	**-7.33** ***				
**Hachioji**	**-6.80** ***	**-7.42** ***	-0.10			
Konan	**-4.80** ***	**-5.42** ***	**1.90** *	**2.00** *		
**Tokamachi**	**-6.68** ***	**-7.31** ***	0.02	0.12	**1.88** *	
**Ikoma**	**-7.42** ***	**-8.05** ***	-0.73	-0.63	**-2.63** **	-0.75

The sign of the significance is indicated as

* *p*<0.05

** *p*<0.01

*** *p*<0.001.

## Discussion

### Middle and late Miocene paleo-[CO_2_]_atm_ change

**Fig 4:**
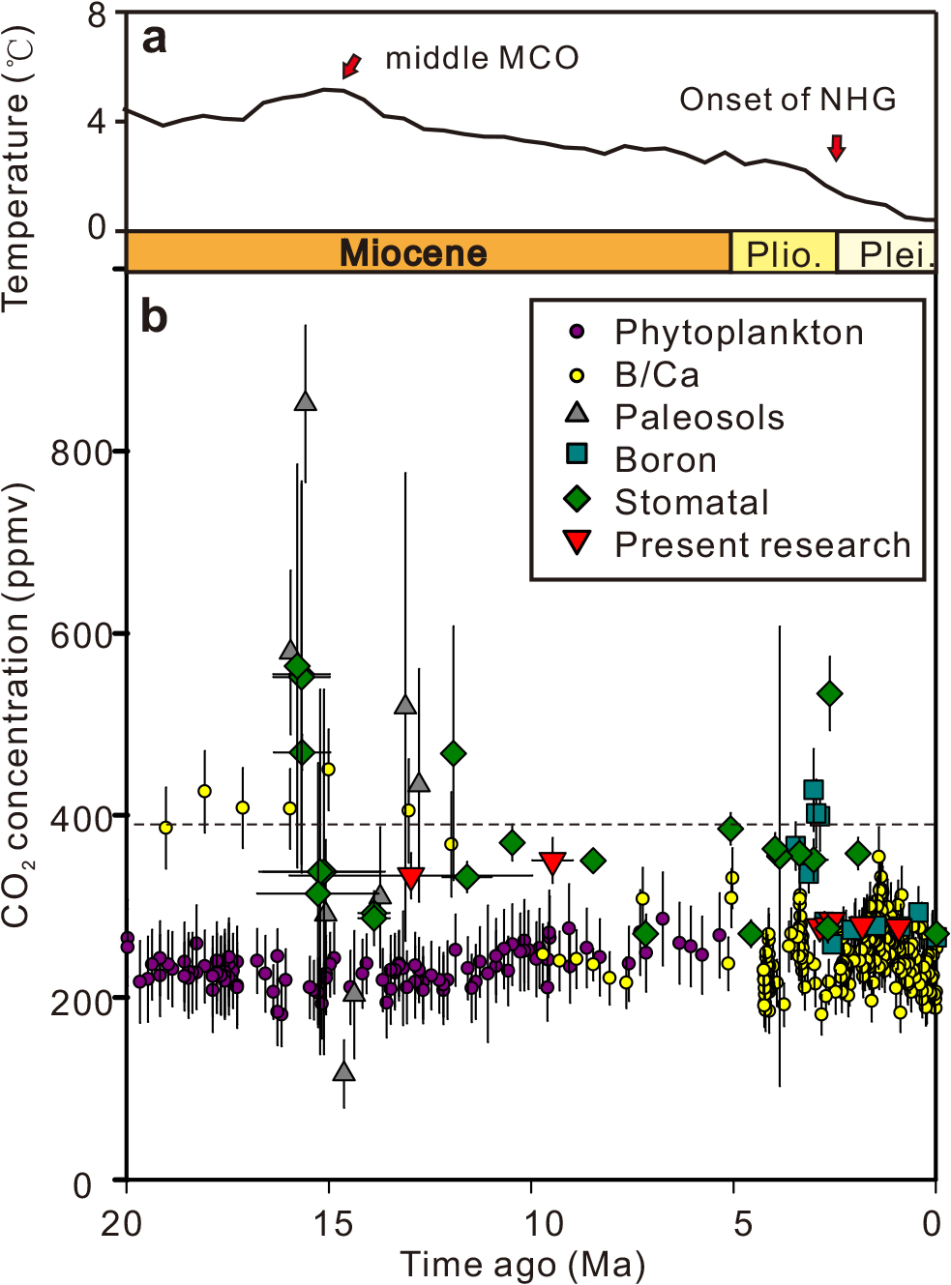
Trend of paleo-[CO_2_]_atm_ during late Cenozoic. (a) Deep-sea temperatures estimated from δ^18^O since 20 Ma [46]; (b) atmospheric CO_**2**_ reconstructed from terrestrial and marine proxies following recent revisions (S2 Table). Vertical error bars: standard deviation of paleo-[CO_**2**_]_**atm**_ values, and horizontal error bars: standard deviation of materials’ age. The current atmospheric CO_**2**_ concentration (390 ppmv) is indicated by the horizontal dashed line.

The paleo-[CO_2_]_atm_ changes reconstructed in previous research generally indicate a peak during the middle Miocene Climatic Optimum (MCO; 17–15 Ma) [44] and a decline during the later stage of the middle Miocene (ca. 15–11.5Ma), although the reconstructed paleo-[CO_2_]_atm_ values and timing of fluctuation were different among proxies (Fig 4B). The most prominent fluctuation was exhibited in the paleosol carbonate records, which showed a spike (ca. 800 ppmv) at 15.6 Ma, drop to 116–310 ppmv at 14.7–13.8 Ma, and increase to 433–519 ppmv around 12.8–13.1 Ma [45]. The stomatal records from fossilized *Quercus* leaves [23] also indicated a prominent change from the highest value (469–555 ppmv) at 15.7±0.7Ma to the lower value at 13.0 Ma (ca.290 ppmv) and 11.6 Ma (ca.330 ppmv) during the late middle Miocene. Additionally, the stomatal proxies from North America indicate lower paleo-[CO_2_]_atm_ values and moderate changes during the earlier stage of the middle Miocene: 396 ppmv from *Ginkgo* leaves at ca. 16.5 Ma and 310–316 ppmv from *Metasequoia* needles around 15.2–15.3 Ma [14].

In general, the values of the middle Miocene [CO_2_]_atm_ estimated from marine proxies are lower than those from terrestrial records. Boron/Calcium (B/Ca) ratios of surface-dwelling foraminifera give a paleo-[CO_2_]_atm_ of ca. 420 ppmv during the MCO that declined gradually to ca. 200 ppmv in the earliest late Miocene [47]. B isotope (δ^11^B)-based paleo-[CO_2_]_atm_ from ODP761 changed from ca. 400 ppmv in the MCO to ca. 280 ppmv in the late middle Miocene [48]. A stable paleo-[CO_2_]_atm_ curve with slight changes around 210 ppmv from the MCO to late Miocene was drawn based on phytoplankton δ^13^C alkene analysis [49,50]. The paleo-[CO_2_]_atm_ value (334 ppmv) reconstructed from the fossilized leaves of the middle Miocene Sanzhangtian site was similar to the late middle Miocene values based on *Quercus* leaves [51] and between the results based on *Ginkgo* (16.5 Ma) and *Metasequoia* (15.2–15.3 Ma) leaves in the early middle Miocene [14].

The late Miocene stomatal data based on fossilized *Quercus* exhibited a decreasing paleo-[CO_2_]_atm_ tendency: ca. 370 ppmv at ca. 10.5 Ma, ca. 350 ppmv at ca. 8.5 Ma, and ca. 270 ppmv at ca. 7.2 Ma [51]. This was related to climatic cooling in the later late Miocene [18,52]. When using B/Ca [53] and phytoplankton [49,50] from marine proxies, they showed fluctuating values that were mostly less than 300 ppmv (Fig 4B). The estimated paleo-[CO_2_]_atm_ values for 10–9 Ma (351 ppmv) from this work are almost the same as the value from ca. 8.5 Ma from *Quercus* leaves [18]. Our data showed little change between the middle Miocene (334 ppmv) and the early late Miocene (351 ppmv) that confirmed the stable paleo-[CO_2_]_atm_ condition during this time as indicated by the phytoplankton record [49,50].

### Late Pliocene to Pleistocene paleo-[CO_2_]_atm_ change

**Fig 5:**
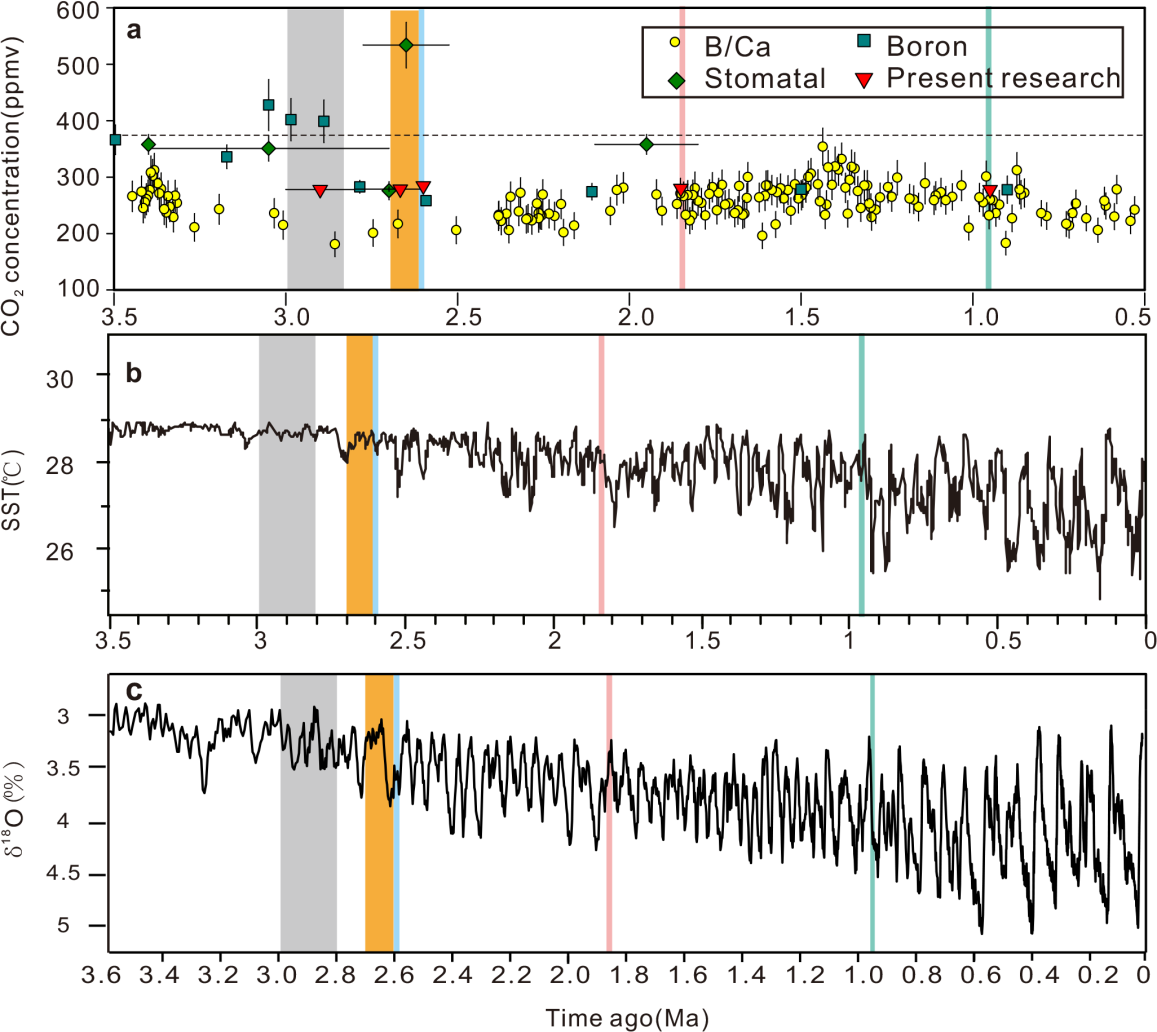
Reconstructed paleo-[CO_2_]_atm_ of Pliocene to Pleistocene compared with reconstructed paleo-temperature and benthic δ^18^O record. (a) Reconstructed paleo-[CO_**2**_]_**atm**_ based on terrestrial and marine proxies following recent revisions (S2 Table) along with our data. Vertical error bars: standard deviation of paleo-[CO_**2**_]_**atm**_ values, and horizontal error bars: standard deviation of ages of materials. The current atmospheric CO_**2**_ concentration (390 ppmv) is indicated by the horizontal dashed line. (b) SST records for the last 3.5 Ma from southern South China Sea [56]. (c) Global oxygen isotopes of benthic foraminifera shells [57]. The vertical color bands in (a), (b), and (c) indicate the periods considered by this research, and same period is marked by the same color.

In most of the previous research, paleo-[CO_2_]_atm_ values are distributed between 200 and 400 ppmv during the Pliocene to Pleistocene (Fig 5A). B/Ca and B data have been used to determine the paleo-[CO_2_]_atm_ of this period, as there is a lack of data from the stomatal method. The paleo-[CO_2_]_atm_ curve based on B/Ca from surface-dwelling foraminifera exhibited a peak of ca. 300 ppmv at ca. 3.4 Ma, this decreased to 181 ppmv at ca. 2.9 Ma, and then increased to 332 ppmv at ca. 1.4 Ma [47]. The downward shift in its fluctuation range was observed in the Early Pleistocene (Fig 5A), and the lowest value of 188 ppmv was recorded in the last glacial maximum (0.02Ma) [47]. The paleo-[CO_2_]_atm_ recorded in the B isotopes indicates a higher level than that in B/Ca record during the Late Pliocene, that is, ca. 340 ppmv at ca. 3.4 Ma and ca. 400 ppmv at ca. 3.0 Ma [54]. However, it decreases to the same level (ca. 270 ppmv) as the B/Ca record in the late Late Pliocene and Early Pleistocene (ca. 2.8–1.0 Ma) [54]. The paleo-[CO_2_]_atm_ level estimated from *Quercus* [18] and *Cupressaceae* [55] stomata indicates a higher level (ca. 350 ppmv) during the early Late Pliocene (3–3.4 Ma) and a lower value (276 ppmv) at 2.7 Ma. While paleo-[CO_2_]_atm_ based on the SI of *Typha* at the Plio-Pleistocene boundary (2.65 Ma) exhibits a much higher value (534 ppmv) than the other results [22].

Our data showed that the paleo-[CO_2_]_atm_ was maintained in the range between 280 and 285 ppmv in the Pliocene and Pleistocene (Figs 4B and 5A), which is about 150 ppmv lower than the results estimated from B isotopes [54], and about 70 ppmv higher than the results estimated from the B/Ca proxy [47]. Our data are consistent with the results estimated from *Quercus* stomata [18], but are much lower than the data estimated from *Typha* from sediment at the Plio-Pleistocene boundary [22]. While different proxies [22,47,54] have recorded fluctuations accompanying climate changes (Fig 5B), the paleo-[CO_2_]_atm_ value of this study stabilized at around 280 ppmv. Seiki et al. concluded that the Pliocene CO_2_ levels determined by numerous methods agreed well with each other [9,54]. The present research suggests that some disagreements still remain in the results between our stomatal data and B, B/Ca records in the Pliocene, while the Pleistocene proxies give more consistent CO_2_ levels (than the Pliocene).

### Paleo-[CO_2_]_atm_ change and late Cenozoic climatic deterioration

The overall climate cooling reconstructed for the past 20 Ma has generally been attributed to changes in CO_2_ concentration in the atmosphere [46,58]. According to the marine oxygen isotope record, global temperature peaked at around 16 Ma (middle MCO) (Fig 4A), and the later part of the middle Miocene is characterized by climate cooling with expansion of the East Arctic ice sheet [59,60]. However, the middle Miocene paleo-[CO_2_]_atm_ reconstructed in this study (around 334 ppmv) was just slightly lower than the present level, which was also the level maintained during the late Miocene (around 354 ppmv). That means that before the global temperature decrease, paleo-[CO_2_]_atm_ had already achieved a stable low level. The Miocene paleo-[CO_2_]_atm_ estimated based on alkenones also showed that paleo-[CO_2_]_atm_ was similar during middle Miocene and late Miocene [61]. The δ^13^C record from foraminifera and B/Ca ratios in the foraminifera suggest that paleo-[CO_2_]_atm_ decreases were apparently synchronous with major episodes of glacial expansion during the middle Miocene [53,62, 63], but this synchronization was not observed in our data. This study supports the view that Miocene climate change was not only influenced by paleo-[CO_2_]_atm_ changes, but also by increases in seasonality and ocean circulation changes [50,64,65], and these accelerated the cooling in the late middle Miocene that also acted to decrease the paleo-[CO_2_]_atm_ [62]. Also, climate sensitivity to paleo-[CO_2_]_atm_ may have been greater than previously thought [66]. The impact of high latitude vegetation on Earth’s albedo may have also played an important role in the Earth’s energy budget in the Miocene [67].

After termination of the mid-Pliocene warmth at ca. 2.9 Ma, cooling trends continued until the onset of major expansion of the Northern Hemisphere ice sheet at ca. 2.7 Ma, which culminated at ca. 2.5 Ma in the earliest Pleistocene [68–70]. However, present results show that the lower paleo-[CO_2_]_atm_ level started around 2.8–3.0 Ma and lasted until the late Early Pleistocene. Therefore, we consider that the transition to the icehouse world was possibly induced by a decrease of the paleo-[CO_2_]_atm_, which already dropped to their lowest levels during the complete Cenozoic before the major expansion of the Northern Hemisphere ice sheets. During the Pliocene to Pleistocene, our data are very stable, but the global temperature estimated from the marine oxygen isotope record [56,57] shows drastic fluctuations (Fig 5). However, our middle and late Miocene data are significantly higher than our Pliocene and Pleistocene data. The oxygen isotope record confirms that the temperature in the Pliocene and Pleistocene was much lower than that of the middle and late Miocene [44,46]. Therefore, we can conclude that the decrease of paleo-[CO_2_]_atm_ level is coupled with temperature decrease during middle Miocene to Pleistocene.

## Conclusions

We used the stomatal index of *Metasequoia* Miki *ex* Hu *et* Cheng as a proxy to reconstruct the paleo-[CO_2_]_atm_ evolution from the middle Miocene to late Early Pleistocene for the first time. Our results indicate that: (1) From middle to late Miocene the atmospheric CO_2_ level stabilized around 350 ppmv which is slightly lower than today. (2) The CO_2_ level during the Pliocene to Pleistocene was similar to the pre-industrial level and no fluctuation can be detected by this research. (3) The Pleistocene CO_2_ level estimated by different proxies agree well with each other. (4) From middle Miocene to Pleistocene, when the global temperature decreased sharply, the global CO_2_ level decreased by more than 50 ppmv, which may suggest that CO_2_ decrease and temperature decrease are coupled.

## Supporting Information

S1 Table Original paleo-[CO_2_]_atm_ results for the seven localities used in this study. (DOC)

S2 Table Previously reconstructed paleo-[CO_2_]_atm_ results based on different proxies over the past 20 Ma. (DOC)

## References

[1] Lacis AA, Schmidt GA, Rind D, Ruedy RA. Atmospheric CO_2_: principal control knob governing Earth's temperature. Science. 2010; 330: 356–359. 10.1126/science.1190653 20947761

[2] Stocker TF, Qin D, Plattner GK, Tignor MMB, Allen SK, Boschung J et al Climate Change 2013: The Physical Science Basis Contribution of Working Group I to the Fifth Assessment Report of the Intergovernmental Panel on Climate Change. 1st ed. Cambridge, United Kingdom and New York, NY, USA: Cambridge University Press; 2013.

[3] Raupach MR, Canadell JG. Carbon and the Anthropocene. Curr Opin Environ Sustain. 2010; 2: 210–218.

[4] Peng J, Dan L, Huang M. Sensitivity of global and regional terrestrial carbon storage to the direct CO_2_ effect and climate change based on the CMIP5 model intercomparison. PLoS ONE. 2014; 9: e95282 10.1371/journal.pone.0095282 24748331 PMC3991598

[5] Berner RA. GEOCARB II: a revised model of atmospheric CO_2_ over Phanerozoic time. American Journal of Science. 1994; 294: 56–91. 10.2475/ajs.289.4.333 11539776

[6] Berner RA. GEOCARBSULF: A combined model for Phanerozoic atmospheric O_2_ and CO_2_ . Geochim Cosmochim Acta. 2006; 70: 5653–5664.

[7] Berner RA, Kothavala Z. GEOCARB III: A revised model of atmospheric CO_2_ over phanerozoic time. American Journal of Science. 2001; 301: 182–204.

[8] Monnin E, Indermühle A, Dällenbach A, Flückiger J, Stauffer B, Stocker TF et al Atmospheric CO_2_ Concentrations over the Last Glacial Termination. Science. 2001; 291: 112–114. 11141559 10.1126/science.291.5501.112

[9] Beerling DJ, Royer DL. Convergent Cenozoic CO_2_ history. Nat Geosci. 2011; 4: 418–420.

[10] Royer DL. CO_2_-forced climate thresholds during the Phanerozoic. Geochim Cosmochim Acta. 2006; 70: 5665–5675.

[11] Solomon S, Qin D, Martin M, Marquis M, Averyt K, Tignor MMB et al Climate Change 2007: The Physical Science Basis. Contribution of Working Group I to the Forth Assessment Report of the Intergovernmental Panel on Climate Change. 1st ed. Cambridge, United Kingdom and New York, NY, USA: Cambridge University Press; 2007.

[12] Doria G, Royer DL, Wolfe AP, Fox A, Westgate JA, Beerling D. Declining Atmospheric CO_2_ during the Late Middle Eocene Climate Transition. American Journal of Science. 2011; 311: 63–75.

[13] Beerling DJ. Stomatal density and index: Theory and application In: Jones TP, Rowe NP, editors. Fossil plants and spores: modern techniques. London: Geological Society of London; 1999 pp. 251–256.

[14] Royer DL, Wing SL, Beerling DJ, Jolley DW, Koch PL, Hickey L et al Paleobotanical evidence for near present-day levels of atmospheric CO_2_ during part of the tertiary. Science. 2001; 292: 2310–2313. 11423657 10.1126/science.292.5525.2310

[15] Quan C, Sun CL, Sun YW, Sun G. High resolution estimates of paleo-CO_2_ levels through the Campanian (Late Cretaceous) based on *Ginkgo* cuticles. Cretaceous Research. 2009; 30: 424–428.

[16] Smith RY, Greenwood DR, Basinger JF. Estimating paleoatmospheric *p*CO_2_ during the Early Eocene Climatic Optimum from stomatal frequency of *Ginkgo*, Okanagan Highlands, British Columbia, Canada. Palaeogeogr Palaeoclimatol Palaeoecol. 2010; 293: 120–131.

[17] Grein M, Oehm C, Konrad W, Utescher T, Kunzmann L, Roth-Nebelsick A. Atmospheric CO_2_ from the late Oligocene to early Miocene based on photosynthesis data and fossil leaf characteristics. Palaeogeogr Palaeoclimatol Palaeoecol. 2013; 374: 41–51.

[18] Kürschner WM, Burgh J, Visscher H, Dilcher DL. Oak leaves as biosensors of late Neogene and early Pleistocene paleoatmospheric CO_2_ concentrations. Mar Micropaleontol, 1996; 27: 299–312.

[19] Sun B, Ding S, Wu J, Dong C. Carbon isotope and stomatal data of Late Pliocene *Betulaceae* leaves from SW China: implications for palaeoatmospheric CO_2_-levels. Turkish Journal of Earth Sciences, 2012; 21: 237–250.

[20] Wagner F, Bohncke SJ, Dilcher DL, Kürschner WM, Geel B, Visscher H. Century-scale shifts in early Holocene atmospheric CO_2_ concentration. Science. 1999; 284: 1971–1973. 10373111 10.1126/science.284.5422.1971

[21] Roth-Nebelsick A, Oehm C, Grein M, Utescher T, Kunzmann L, Friedrich JP et al Stomatal density and index data of *Platanus neptuni* leaf fossils and their evaluation as a CO_2_ proxy for the Oligocene. Rev Palaeobot Palynol. 2014; 206: 1–9.

[22] Bai YJ, Chen LQ, Ranhotra PS, Wang Q, Wang YF, Li CS. Reconstructing atmospheric CO_2_ during the Plio-Pleistocene transition by fossil *Typha* . Glob Chang Biol. 2014; 21: 874–881. 10.1111/gcb.12670 24990109

[23] Kürschner WM, Kvacek Z, Dilcher DL. The impact of Miocene atmospheric carbon dioxide fluctuations on climate and the evolution of terrestrial ecosystems. PNAS. 2008; 105: 449–453. 10.1073/pnas.0708588105 18174330 PMC2206556

[24] Royer DL. Stomatal density and stomatal index as indicators of paleoatmospheric CO_2_ concentration. Rev Palaeobot Palynol. 2001; 114: 1–28. 11295163 10.1016/s0034-6667(00)00074-9

[25] Basinger JF. The Vegetative Body of *Metasequoia-Milleri* from the Middle Eocene of Southern British-Columbia. Can J Bot. 1981; 59: 2379–2410.

[26] LePage BA, Williams CJ, Yang H. The geobiology and ecology of *Metasequoia* Dordrecht: Springer; 2005.

[27] Ge HR, Li DY. Cenozoic coal-bearing basins and coal-forming regularity in West Yunnan Kunming: Yunnan Science and Technology Press; 1999.

[28] Zhang QQ, Ferguson DK, Mosbrugger V, Wang YF, Li CS. Vegetation and climatic changes of SW China in response to the uplift of Tibetan Plateau. Palaeogeogr Palaeoclimatol Palaeoecol. 2012; 363: 23–36.

[29] Wang L, Zhou ZK, Xing YW, Su T, Jacques FMB, Liu YS. Miocene *Metasequoia* from Yunnan, southwest China and its biological implications. Japanese Journal of Palynology (Special Issue: Abstract Issue for the Joint Meeting of 13^th^ International Palynological Congress and 9th International Organisation of Palaeobotany Conference). 2012; 58: 251.

[30] Kobayashi M., Saito T., Okitsu S. Zircon fission-track ages of the Miocene Yagii Formation, Saitama Prefecture, central Japan, and their palaeoecological significance. The journal of the Geological Society of Japan. 2011; 117 632–636.

[31] Itihara M, Ichikawa K, Yamada N. Geology of the Kishiwada district with geological sheet map at 1:50,000 Tsukuba: Geological Survey of Japan; 1986.

[32] Satoguchi Y, Nagahashi Y. Tephrostratigraphy of the Pliocene to Middle Pleistocene Series in Honshu and Kyushu Islands, Japan. Island Arc. 2012; 21: 149–169.

[33] Tomita Y, Kurokawa K. A widespread volcanic ash layer of about 2.7 Ma in central Japan: correlation of the Habutaki I (Osaka Group), the MT2 (Himi Group) and the Arg-2 (Nishiyama Formation) ash layers. Journal of the Geological Society of Japan. 1999; 105: 63–71.

[34] Horiuchi J. Neogene Flora of the Kanto District. Science Reports of the Institute of Geoscience Geological Sciences, Tsukuba University, Section B, Geological Sciences. 1996; 17: 109–208.

[35] Kimura T, Ohana T, Yoshiyama H. Fossil plants from the Tama and Azuyama Hills, Southern Kwanto, Japan. Transactions and Proceedings of the Paleontological Society of Japan. 1984; 122: 87–104.

[36] Satoguchi Y, Yamakawa C and Takahashi K. The old and newly defined Pliocene-Pleistocene boundary sites of the Kobiwako Group, central Japan. Journal of Geological Society. 2012; 118: 70–78.

[37] Niigata Fossil Plant Research Group and Niigata Pollen Research Group. Plant megafossils and pollen fossils from the Uonuma Group, Niigata Prefecture, central Japan In: Uonuma Hills Collaborative Research Group, editor. The Uonuma Group. Tokyo: The Association for the Geological Collaboration in Japan; 1983.

[38] Yanagisawa Y, Kayahara K, Suzuki Y, Uemura T, Kodama K, Kato T. Geology of the Tokamachi District with geological sheet map at 1:50,000 Tsukuba: Geological Survey of Japan; 1985.

[39] Mitamura M. Stratigraphy and Geologic Structure of the Osaka Group (Pliocene and Pleistocene) in Keihanna Hills, Kinki District, Japan. The Quaternary Research. 1992; 31: 159–177.

[40] Yoshikawa S, Mitamura M. Quaternary stratigraphy of the Osaka Plain, central Japan and its correlation with oxygen isotope record from deep sea cores. The Geological Society of Japan. 1999; 105: 332–340.

[41] Kerp H. The study of fossil gymnosperms by means of cuticular analysis. Palaios. 1990; 5: 548–569.

[42] Jones TP, Rowe NP, editors. Fossil plants and spores: modern techniques London: Geological Society of London; 1999.

[43] Salisbury EJ. On the causes and ecological significance of stomatal frequency, with special reference to the woodland flora In: Philosophical Transactions of the Royal Society of London (vol. 216). London: The Royal Society; 1982 pp. 1–65.

[44] Zachos J, Pagani M, Sloan LC, Thomas E, Billups K. Trends, rhythms, and aberrations in global climate 65 Ma to present. Science. 2001; 292: 686–693. 11326091 10.1126/science.1059412

[45] Retallack GJ. Refining a pedogenic-carbonate CO_2_ paleobarometer to quantify a middle Miocene greenhouse spike. Palaeogeogr Palaeoclimatol Palaeoecol. 2009; 281: 57–65.

[46] Zachos JC, Dickens GR, Zeebe RE. An early Cenozoic perspective on greenhouse warming and carbon-cycle dynamics. Nature. 2008; 451: 279–283. 10.1038/nature06588 18202643

[47] Tripati AK, Roberts CD, Eagle RA, Li G (2011) A 20 million year record of planktic foraminiferal B/Ca ratios: Systematics and uncertainties in *p*CO_2_ reconstructions. Geochim Cosmochim Acta. 2011; 75: 2582–2610.

[48] Foster GL, Lear CH, Rae JW. The evolution of *p*CO_2_, ice volume and climate during the middle Miocene. Earth Planet Sci Lett. 2012; 341: 243–254.

[49] Henderiks J, Pagani M. Coccolithophore cell size and the Paleogene decline in atmospheric CO_2_ . Earth Planet Sci Lett. 2008; 269: 576–584.

[50] Pagani M, Lemarchand D, Spivack A, Gaillardet J. A critical evaluation of the boron isotope-pH proxy: The accuracy of ancient ocean pH estimates. Geochim Cosmochim Acta. 2005; 69: 953–961.

[51] Kürschner WM. Leaf stomata as biosensors of paleoatmospheric CO_2_ levels. PhD Thesis, Utrecht University. 1996. Available: http://library.wur.nl/WebQuery/clc/94370.

[52] Burgh J, Visscher H, Dilcher DL, Kurschner WM. Paleoatmospheric signatures in Neogene fossil leaves. Science. 1993; 260: 1788–1790. 17793657 10.1126/science.260.5115.1788

[53] Tripati AK, Roberts CD, Eagle RA. Coupling of CO_2_ and ice sheet stability over major climate transitions of the last 20 million years. Science. 2009; 326: 1394–1397. 10.1126/science.1178296 19815724

[54] Seki O, Foster GL, Schmidt DN, Mackensen A, Kawamura K, Pancost RD. Alkenone and Boron-based Pliocene *p*CO_2_ records. Earth Planet Sci Lett. 2010; 292: 201–211.

[55] Stults DZ, Wagner-Cremer F, Axsmith BJ. Atmospheric paleo-CO_2_ estimates based on *Taxodium distichum* (Cupressaceae) fossils from the Miocene and Pliocene of Eastern North America. Palaeogeogr Palaeoclimatol Palaeoecol. 2011; 309: 327–332.

[56] Li L, Li Q, Tian J, Wang P, Wang H, Liu ZH. A 4-Ma record of thermal evolution in the tropical western Pacific and its implications on climate change. Earth Planet Sci Lett. 2011; 309: 10–20.

[57] Lisiecki LE, Raymo ME. A Pliocene-Pleistocene stack of 57 globally distributed benthic δ^18^O records. Paleoceanography. 2005; 20: PA1003.

[58] Ruddiman WF. Orbital insolation, ice volume, and greenhouse gases. Quat Sci Rev. 2003; 22: 1597–1629.

[59] Flower BP, Kennett JP. The middle Miocene climatic transition: East Antarctic ice sheet development, deep ocean circulation and global carbon cycling. Palaeogeogr Palaeoclimatol Palaeoecol. 1994; 108: 537–555.

[60] Shevenell AE, Kennett JP, Lea DW. Middle Miocene ice sheet dynamics, deep-sea temperatures, and carbon cycling: A Southern Ocean perspective. Geochemistry, Geophysics, Geosystems. 2008; 9: Q02006.

[61] Zhang YG, Pagani M, Liu Z, Bohaty SM, DeConto R. (2013) A 40-million-year history of atmospheric CO_2_ . 2013; 373: 1–20. 10.1098/rsta.2013.0096 24043869

[62] Vincent E, Berger WH. Carbon dioxide and polar cooling in the Miocene: The Monterey hypothesis. The Carbon cycle and atmospheric CO_2_: Natural Variations Archean to Present. 1985; 32: 455–468.

[63] Badger MPS, Lear CH, Pancost RD, Foster GL, Bailey TR, Leng MG et al CO_2_ drawdown following the middle Miocene expansion of the Antarctic Ice Sheet. Paleoceanography. 2013; 28: 12.

[64] Mosbrugger V, Utescher T, Dilcher DL. Cenozoic continental climatic evolution of Central Europe. PNAS. 2005; 102: 14964–14969. 16217023 10.1073/pnas.0505267102 PMC1257711

[65] Shevenell AE, Kennett JP, Lea DW. Middle Miocene Southern Ocean cooling and Antarctic cryosphere expansion. Science. 2004; 305: 1766–1770. 15375266 10.1126/science.1100061

[66] Pearson PN, Ditchfield PW, Singano J, Harcourt-Brown KG, Nicholas CJ, Olsson RK et al Warm tropical sea surface temperatures in the Late Cretaceous and Eocene epochs. Nature. 2001; 414: 470–470. 10.1038/35097000 11586350

[67] Knorr G, Butzin M, Micheels A, Lohmann G. A warm Miocene climate at low atmospheric CO_2_ levels. Geophys Res Lett. 2011; 38: 5.

[68] Schepper SD, Groeneveld J, Naafs BDA, Renterghem CV, Hennissen J, Head MJ. Northern hemisphere glaciation during the globally warm early late Pliocene. PLoS ONE. 2013; 8: e81508 10.1371/journal.pone.0081508 24349081 PMC3861316

[69] Domingo L, Koch PL, Fernández HM, Fox DL, Domingo MS, Alberdi MT et al Late Neogene and early Quaternary paleoenvironmental and paleoclimatic Conditions in Southwestern Europe: Isotopic analyses on mammalian taxa. PLoS ONE. 2013; 8: e63739 10.1371/journal.pone.0063739 23717470 PMC3662777

[70] Raymo M. The initiation of Northern Hemisphere glaciation. Annual Review of Earth and Planetary Sciences. 1994; 22: 353–383.

