# Supplementary material for: Evolutionary History of Atmospheric CO2 during the Late Cenozoic from Fossilized Metasequoia Needles
Source: PLoS One. 2015 Jul 8;10(7):e0130941. doi: 10.1371/journal.pone.0130941 (PMC4511968; doi:10.1371/journal.pone.0130941)
Supplement: S1 Table — (DOC) [file pone.0130941.s001.doc]

| Locality | Sample # | Epidermis cell (No.) | Stoma (No.) | SI | CO_2_ |
| --- | --- | --- | --- | --- | --- |
| Sanzhangtian | SZT077 | 126 | 18 | 12.50 | 297.84 |
| Sanzhangtian | SZT077 | 115 | 16 | 12.21 | 300.27 |
| Sanzhangtian | SZT077 | 94 | 12 | 11.32 | 310.15 |
| Sanzhangtian | SZT077 | 123 | 13 | 9.56 | 354.35 |
| Sanzhangtian | SZT077 | 127 | 13 | 9.29 | 368.83 |
| Sanzhangtian | SZT077 | 127 | 15 | 10.56 | 323.00 |
| Sanzhangtian | SZT077 | 201 | 24 | 10.67 | 320.89 |
| Sanzhangtian | SZT077 | 110 | 12 | 9.84 | 343.05 |
| Sanzhangtian | SZT077 | 100 | 10 | 9.09 | 382.13 |
| Sanzhangtian | SZT077 | 136 | 18 | 11.69 | 305.58 |
| Sanzhangtian | SZT077 | 205 | 22 | 9.69 | 348.58 |
| Sanzhangtian | SZT077 | 147 | 16 | 9.82 | 343.78 |
| Sanzhangtian | SZT077 | 145 | 15 | 9.38 | 363.64 |
| Sanzhangtian | SZT077 | 79 | 9 | 10.23 | 330.95 |
| Sanzhangtian | SZT077 | 79 | 9 | 10.23 | 330.95 |
| Sanzhangtian | SZT077 | 132 | 15 | 10.20 | 331.57 |
| Sanzhangtian | SZT077 | 115 | 15 | 11.54 | 307.35 |
| Sanzhangtian | SZT077 | 71 | 9 | 11.25 | 311.14 |
| Sanzhangtian | SZT077 | 78 | 9 | 10.34 | 327.96 |
| Sanzhangtian | SZT115 | 128 | 13 | 9.22 | 373.00 |
| Sanzhangtian | SZT123 | 111 | 13 | 10.48 | 324.72 |
| Sanzhangtian | SZT077 | 104 | 14 | 11.86 | 303.66 |
| Sanzhangtian | SZT127 | 161 | 19 | 10.56 | 323.17 |
| Sanzhangtian | SZT156 | 136 | 15 | 9.93 | 339.67 |
| Sanzhangtian | SZT126 | 79 | 8 | 9.20 | 374.63 |
| Kumagaya | YJ003 | 379 | 38 | 9.11 | 380.49 |
| Kumagaya | YJ003 | 195 | 24 | 10.96 | 315.60 |
| Kumagaya | YJ003 | 119 | 14 | 10.53 | 323.79 |
| Kumagaya | YJ003 | 295 | 34 | 10.33 | 328.22 |
| Kumagaya | YJ003 | 136 | 14 | 9.33 | 366.00 |
| Kumagaya | YJ003 | 78 | 9 | 10.34 | 327.96 |
| Kumagaya | YJ003 | 646 | 67 | 9.40 | 362.44 |
| Kumagaya | YJ005 | 321 | 35 | 9.83 | 343.21 |
| Kumagaya | YJ005 | 226 | 24 | 9.60 | 352.48 |
| Kumagaya | YJ005 | 251 | 26 | 9.39 | 363.02 |
| Kumagaya | YJ005 | 142 | 14 | 8.97 | 391.73 |
| Sennan | FT001 | 357 | 88 | 19.78 | 274.03 |
| Sennan | FT001 | 404 | 98 | 19.52 | 274.37 |
| Sennan | FT001 | 423 | 77 | 15.40 | 283.12 |
| Sennan | FT001 | 378 | 67 | 15.06 | 284.28 |
| Sennan | FT001 | 279 | 62 | 18.18 | 276.48 |
| Sennan | FT001 | 276 | 48 | 14.81 | 285.15 |
| Hachioji | BQC001 | 228 | 43 | 15.87 | 281.70 |
| Hachioji | BQC001 | 167 | 37 | 18.14 | 276.56 |
| Hachioji | BQC001 | 180 | 37 | 17.05 | 278.71 |
| Hachioji | BQC001 | 263 | 60 | 18.58 | 275.81 |
| Hachioji | BQC001 | 454 | 79 | 14.82 | 285.13 |
| Hachioji | BQC001 | 380 | 85 | 18.28 | 276.31 |
| Hachioji | BQC001 | 271 | 65 | 19.35 | 274.62 |
| Hachioji | BQC001 | 242 | 45 | 15.68 | 282.25 |
| Konan | SG001 | 155 | 35 | 18.42 | 276.07 |
| Konan | SG001 | 232 | 41 | 15.02 | 284.41 |
| Konan | SG001 | 212 | 42 | 16.54 | 279.92 |
| Konan | SG001 | 253 | 40 | 13.65 | 290.34 |
| Konan | SG001 | 232 | 37 | 13.75 | 289.81 |
| Konan | SG002 | 151 | 26 | 14.69 | 285.63 |
| Konan | SG002 | 301 | 51 | 14.49 | 286.44 |
| Tokamachi | 156u01 | 203 | 31 | 13.25 | 292.63 |
| Tokamachi | 156u01 | 126 | 27 | 17.65 | 277.47 |
| Tokamachi | 156u01 | 91 | 19 | 17.27 | 278.23 |
| Tokamachi | 156u01 | 86 | 17 | 16.50 | 279.99 |
| Tokamachi | 156u01 | 152 | 27 | 15.08 | 284.18 |
| Tokamachi | 156u01 | 144 | 40 | 21.74 | 271.76 |
| Tokamachi | 156u01 | 256 | 46 | 15.23 | 283.67 |
| Tokamachi | 156u01 | 334 | 56 | 14.36 | 286.98 |
| Tokamachi | 156u01 | 271 | 68 | 20.06 | 273.66 |
| Tokamachi | 156u01 | 248 | 50 | 16.78 | 279.33 |
| Tokamachi | 156u01 | 266 | 71 | 21.07 | 272.46 |
| Tokamachi | 156u01 | 386 | 101 | 20.74 | 272.83 |
| Tokamachi | 156u01 | 288 | 49 | 14.54 | 286.23 |
| Tokamachi | 156u01 | 253 | 56 | 18.12 | 276.58 |
| Tokamachi | 156u01 | 382 | 78 | 16.96 | 278.92 |
| Tokamachi | 156u01 | 243 | 45 | 15.63 | 282.41 |
| Tokamachi | 156u01 | 266 | 50 | 15.82 | 281.82 |
| Ikoma | NR001 | 243 | 56 | 18.73 | 275.56 |
| Ikoma | NR001 | 340 | 86 | 20.19 | 273.49 |
| Ikoma | NR001 | 365 | 67 | 15.51 | 282.77 |
| Ikoma | NR001 | 310 | 57 | 15.53 | 282.70 |
| Ikoma | NR001 | 434 | 89 | 17.02 | 278.79 |
| Ikoma | NR001 | 304 | 75 | 19.79 | 274.01 |
| Ikoma | NR001 | 135 | 30 | 18.18 | 276.48 |
