# Supplementary material for: Evolutionary History of Atmospheric CO2 during the Late Cenozoic from Fossilized Metasequoia Needles
Source: PLoS One. 2015 Jul 8;10(7):e0130941. doi: 10.1371/journal.pone.0130941 (PMC4511968; doi:10.1371/journal.pone.0130941)
Supplement: S2 Table — (DOC) [file pone.0130941.s002.doc]

| Method | Study | Age (Ma) | Age_min_ (Ma) | Ag_emax_ (Ma) | CO_2_ (ppmv) | CO_2min_ (ppmv) | CO_2max (_ppmv) |
| --- | --- | --- | --- | --- | --- | --- | --- |
| Boron | [1] | 0.0 | - | - | 266 | 249 | 283 |
| Boron | [1] | 0.5 | - | - | 293 | 265 | 321 |
| Boron | [1] | 0.9 | - | - | 278 | 254 | 302 |
| Boron | [1] | 1.5 | - | - | 279 | 263 | 294 |
| Boron | [1] | 2.1 | - | - | 274 | 260 | 289 |
| Boron | [1] | 2.6 | - | - | 258 | 251 | 266 |
| Boron | [1] | 2.8 | - | - | 283 | 272 | 294 |
| Boron | [1] | 2.9 | - | - | 399 | 361 | 437 |
| Boron | [1] | 3.0 | - | - | 402 | 365 | 439 |
| Boron | [1] | 3.0 | - | - | 428 | 383 | 473 |
| Boron | [1] | 3.2 | - | - | 336 | 315 | 357 |
| Boron | [1] | 3.5 | - | - | 366 | 340 | 393 |
| B/Ca | [2] | 0.006 | - | - | 254 | 229 | 275 |
| B/Ca | [2] | 0.014 | - | - | 206 | 190 | 232 |
| B/Ca | [2] | 0.028 | - | - | 188 | 169 | 208 |
| B/Ca | [2] | 0.037 | - | - | 198 | 179 | 219 |
| B/Ca | [2] | 0.052 | - | - | 203 | 185 | 226 |
| B/Ca | [2] | 0.09 | - | - | 224 | 203 | 246 |
| B/Ca | [2] | 0.091 | - | - | 246 | 224 | 269 |
| B/Ca | [2] | 0.106 | - | - | 270 | 249 | 299 |
| B/Ca | [2] | 0.111 | - | - | 267 | 246 | 296 |
| B/Ca | [2] | 0.128 | - | - | 221 | 200 | 243 |
| B/Ca | [2] | 0.146 | - | - | 203 | 186 | 226 |
| B/Ca | [2] | 0.164 | - | - | 205 | 187 | 228 |
| B/Ca | [2] | 0.198 | - | - | 226 | 207 | 252 |
| B/Ca | [2] | 0.212 | - | - | 246 | 226 | 271 |
| B/Ca | [2] | 0.244 | - | - | 207 | 190 | 232 |
| B/Ca | [2] | 0.247 | - | - | 193 | 177 | 219 |
| B/Ca | [2] | 0.253 | - | - | 242 | 224 | 269 |
| B/Ca | [2] | 0.255 | - | - | 191 | 174 | 215 |
| B/Ca | [2] | 0.259 | - | - | 239 | 222 | 268 |
| B/Ca | [2] | 0.267 | - | - | 189 | 170 | 210 |
| B/Ca | [2] | 0.297 | - | - | 225 | 204 | 247 |
| B/Ca | [2] | 0.316 | - | - | 270 | 252 | 301 |
| B/Ca | [2] | 0.33 | - | - | 233 | 215 | 262 |
| B/Ca | [2] | 0.36 | - | - | 198 | 182 | 222 |
| B/Ca | [2] | 0.367 | - | - | 207 | 187 | 227 |
| B/Ca | [2] | 0.374 | - | - | 279 | 259 | 309 |
| B/Ca | [2] | 0.379 | - | - | 220 | 204 | 247 |
| B/Ca | [2] | 0.424 | - | - | 276 | 257 | 308 |
| B/Ca | [2] | 0.426 | - | - | 214 | 200 | 243 |
| B/Ca | [2] | 0.529 | - | - | 242 | 224 | 271 |
| B/Ca | [2] | 0.541 | - | - | 222 | 204 | 248 |
| B/Ca | [2] | 0.581 | - | - | 278 | 258 | 308 |
| B/Ca | [2] | 0.588 | - | - | 230 | 212 | 258 |
| B/Ca | [2] | 0.613 | - | - | 249 | 227 | 273 |
| B/Ca | [2] | 0.617 | - | - | 258 | 239 | 287 |
| B/Ca | [2] | 0.636 | - | - | 206 | 192 | 235 |
| B/Ca | [2] | 0.67 | - | - | 227 | 210 | 254 |
| B/Ca | [2] | 0.7 | - | - | 253 | 233 | 281 |
| B/Ca | [2] | 0.71 | - | - | 236 | 218 | 264 |
| B/Ca | [2] | 0.72 | - | - | 214 | 198 | 242 |
| B/Ca | [2] | 0.728 | - | - | 217 | 200 | 244 |
| B/Ca | [2] | 0.785 | - | - | 231 | 212 | 259 |
| B/Ca | [2] | 0.801 | - | - | 236 | 219 | 264 |
| B/Ca | [2] | 0.85 | - | - | 272 | 252 | 302 |
| B/Ca | [2] | 0.86 | - | - | 269 | 250 | 300 |
| B/Ca | [2] | 0.864 | - | - | 278 | 259 | 310 |
| B/Ca | [2] | 0.872 | - | - | 312 | 287 | 350 |
| B/Ca | [2] | 0.886 | - | - | 227 | 210 | 257 |
| B/Ca | [2] | 0.904 | - | - | 183 | 167 | 209 |
| B/Ca | [2] | 0.923 | - | - | 251 | 232 | 283 |
| B/Ca | [2] | 0.933 | - | - | 236 | 218 | 264 |
| B/Ca | [2] | 0.942 | - | - | 259 | 241 | 290 |
| B/Ca | [2] | 0.95 | - | - | 260 | 239 | 287 |
| B/Ca | [2] | 0.953 | - | - | 232 | 214 | 261 |
| B/Ca | [2] | 0.961 | - | - | 301 | 280 | 335 |
| B/Ca | [2] | 0.97 | - | - | 255 | 235 | 283 |
| B/Ca | [2] | 0.981 | - | - | 264 | 246 | 296 |
| B/Ca | [2] | 1.012 | - | - | 210 | 191 | 234 |
| B/Ca | [2] | 1.033 | - | - | 285 | 265 | 318 |
| B/Ca | [2] | 1.06 | - | - | 265 | 246 | 293 |
| B/Ca | [2] | 1.079 | - | - | 259 | 239 | 288 |
| B/Ca | [2] | 1.093 | - | - | 273 | 254 | 304 |
| B/Ca | [2] | 1.108 | - | - | 267 | 249 | 299 |
| B/Ca | [2] | 1.113 | - | - | 259 | 241 | 291 |
| B/Ca | [2] | 1.139 | - | - | 289 | 257 | 330 |
| B/Ca | [2] | 1.158 | - | - | 247 | 229 | 278 |
| B/Ca | [2] | 1.172 | - | - | 260 | 240 | 289 |
| B/Ca | [2] | 1.184 | - | - | 262 | 243 | 293 |
| B/Ca | [2] | 1.22 | - | - | 299 | 275 | 336 |
| B/Ca | [2] | 1.236 | - | - | 265 | 247 | 298 |
| B/Ca | [2] | 1.247 | - | - | 282 | 262 | 317 |
| B/Ca | [2] | 1.27 | - | - | 264 | 245 | 296 |
| B/Ca | [2] | 1.283 | - | - | 242 | 221 | 269 |
| B/Ca | [2] | 1.295 | - | - | 229 | 210 | 256 |
| B/Ca | [2] | 1.297 | - | - | 246 | 227 | 276 |
| B/Ca | [2] | 1.302 | - | - | 253 | 235 | 285 |
| B/Ca | [2] | 1.307 | - | - | 285 | 265 | 318 |
| B/Ca | [2] | 1.316 | - | - | 285 | 266 | 319 |
| B/Ca | [2] | 1.326 | - | - | 245 | 225 | 272 |
| B/Ca | [2] | 1.335 | - | - | 275 | 256 | 308 |
| B/Ca | [2] | 1.344 | - | - | 315 | 294 | 351 |
| B/Ca | [2] | 1.353 | - | - | 318 | 295 | 355 |
| B/Ca | [2] | 1.359 | - | - | 295 | 276 | 329 |
| B/Ca | [2] | 1.364 | - | - | 235 | 214 | 261 |
| B/Ca | [2] | 1.371 | - | - | 281 | 261 | 313 |
| B/Ca | [2] | 1.382 | - | - | 332 | 311 | 368 |
| B/Ca | [2] | 1.391 | - | - | 313 | 291 | 346 |
| B/Ca | [2] | 1.401 | - | - | 319 | 291 | 360 |
| B/Ca | [2] | 1.41 | - | - | 286 | 267 | 318 |
| B/Ca | [2] | 1.421 | - | - | 317 | 294 | 354 |
| B/Ca | [2] | 1.426 | - | - | 251 | 234 | 284 |
| B/Ca | [2] | 1.43 | - | - | 233 | 216 | 264 |
| B/Ca | [2] | 1.437 | - | - | 354 | 328 | 394 |
| B/Ca | [2] | 1.445 | - | - | 257 | 239 | 290 |
| B/Ca | [2] | 1.451 | - | - | 287 | 268 | 320 |
| B/Ca | [2] | 1.471 | - | - | 306 | 286 | 340 |
| B/Ca | [2] | 1.479 | - | - | 300 | 279 | 336 |
| B/Ca | [2] | 1.486 | - | - | 281 | 261 | 312 |
| B/Ca | [2] | 1.495 | - | - | 271 | 252 | 301 |
| B/Ca | [2] | 1.499 | - | - | 267 | 247 | 297 |
| B/Ca | [2] | 1.508 | - | - | 262 | 243 | 294 |
| B/Ca | [2] | 1.518 | - | - | 287 | 267 | 320 |
| B/Ca | [2] | 1.53 | - | - | 240 | 221 | 268 |
| B/Ca | [2] | 1.538 | - | - | 277 | 258 | 309 |
| B/Ca | [2] | 1.55 | - | - | 262 | 244 | 293 |
| B/Ca | [2] | 1.56 | - | - | 280 | 262 | 313 |
| B/Ca | [2] | 1.57 | - | - | 273 | 255 | 307 |
| B/Ca | [2] | 1.574 | - | - | 216 | 199 | 245 |
| B/Ca | [2] | 1.585 | - | - | 277 | 259 | 311 |
| B/Ca | [2] | 1.597 | - | - | 285 | 266 | 319 |
| B/Ca | [2] | 1.602 | - | - | 285 | 266 | 318 |
| B/Ca | [2] | 1.605 | - | - | 267 | 248 | 300 |
| B/Ca | [2] | 1.613 | - | - | 196 | 178 | 223 |
| B/Ca | [2] | 1.622 | - | - | 264 | 246 | 297 |
| B/Ca | [2] | 1.655 | - | - | 300 | 281 | 333 |
| B/Ca | [2] | 1.658 | - | - | 263 | 245 | 297 |
| B/Ca | [2] | 1.665 | - | - | 235 | 217 | 265 |
| B/Ca | [2] | 1.67 | - | - | 233 | 214 | 262 |
| B/Ca | [2] | 1.68 | - | - | 284 | 266 | 319 |
| B/Ca | [2] | 1.684 | - | - | 241 | 223 | 272 |
| B/Ca | [2] | 1.693 | - | - | 236 | 219 | 267 |
| B/Ca | [2] | 1.71 | - | - | 253 | 234 | 284 |
| B/Ca | [2] | 1.721 | - | - | 251 | 232 | 282 |
| B/Ca | [2] | 1.73 | - | - | 286 | 268 | 321 |
| B/Ca | [2] | 1.74 | - | - | 272 | 254 | 306 |
| B/Ca | [2] | 1.745 | - | - | 241 | 223 | 271 |
| B/Ca | [2] | 1.758 | - | - | 282 | 263 | 315 |
| B/Ca | [2] | 1.767 | - | - | 270 | 252 | 303 |
| B/Ca | [2] | 1.779 | - | - | 232 | 214 | 262 |
| B/Ca | [2] | 1.79 | - | - | 254 | 235 | 287 |
| B/Ca | [2] | 1.8 | - | - | 258 | 240 | 291 |
| B/Ca | [2] | 1.809 | - | - | 280 | 259 | 317 |
| B/Ca | [2] | 1.815 | - | - | 268 | 249 | 303 |
| B/Ca | [2] | 1.817 | - | - | 232 | 214 | 263 |
| B/Ca | [2] | 1.824 | - | - | 223 | 204 | 250 |
| B/Ca | [2] | 1.83 | - | - | 268 | 250 | 302 |
| B/Ca | [2] | 1.836 | - | - | 233 | 214 | 262 |
| B/Ca | [2] | 1.842 | - | - | 266 | 248 | 299 |
| B/Ca | [2] | 1.851 | - | - | 271 | 252 | 306 |
| B/Ca | [2] | 1.861 | - | - | 252 | 233 | 283 |
| B/Ca | [2] | 1.905 | - | - | 240 | 220 | 269 |
| B/Ca | [2] | 1.921 | - | - | 269 | 249 | 302 |
| B/Ca | [2] | 2.017 | - | - | 281 | 262 | 316 |
| B/Ca | [2] | 2.036 | - | - | 277 | 256 | 309 |
| B/Ca | [2] | 2.055 | - | - | 240 | 220 | 268 |
| B/Ca | [2] | 2.161 | - | - | 214 | 193 | 239 |
| B/Ca | [2] | 2.192 | - | - | 202 | 181 | 227 |
| B/Ca | [2] | 2.199 | - | - | 252 | 231 | 282 |
| B/Ca | [2] | 2.218 | - | - | 231 | 212 | 261 |
| B/Ca | [2] | 2.234 | - | - | 235 | 214 | 264 |
| B/Ca | [2] | 2.252 | - | - | 269 | 247 | 300 |
| B/Ca | [2] | 2.256 | - | - | 232 | 209 | 258 |
| B/Ca | [2] | 2.26 | - | - | 233 | 211 | 261 |
| B/Ca | [2] | 2.264 | - | - | 236 | 210 | 260 |
| B/Ca | [2] | 2.264 | - | - | 227 | 206 | 255 |
| B/Ca | [2] | 2.269 | - | - | 253 | 232 | 283 |
| B/Ca | [2] | 2.284 | - | - | 223 | 200 | 248 |
| B/Ca | [2] | 2.295 | - | - | 227 | 207 | 256 |
| B/Ca | [2] | 2.3 | - | - | 225 | 204 | 252 |
| B/Ca | [2] | 2.317 | - | - | 272 | 250 | 304 |
| B/Ca | [2] | 2.322 | - | - | 239 | 218 | 268 |
| B/Ca | [2] | 2.346 | - | - | 265 | 245 | 298 |
| B/Ca | [2] | 2.351 | - | - | 206 | 188 | 233 |
| B/Ca | [2] | 2.358 | - | - | 235 | 214 | 267 |
| B/Ca | [2] | 2.373 | - | - | 222 | 202 | 250 |
| B/Ca | [2] | 2.38 | - | - | 233 | 212 | 261 |
| B/Ca | [2] | 2.381 | - | - | 231 | 210 | 261 |
| B/Ca | [2] | 2.504 | - | - | 206 | 184 | 232 |
| B/Ca | [2] | 2.674 | - | - | 217 | 194 | 243 |
| B/Ca | [2] | 2.746 | - | - | 201 | 179 | 226 |
| B/Ca | [2] | 2.857 | - | - | 181 | 159 | 203 |
| B/Ca | [2] | 3.008 | - | - | 215 | 192 | 242 |
| B/Ca | [2] | 3.034 | - | - | 236 | 212 | 264 |
| B/Ca | [2] | 3.194 | - | - | 243 | 222 | 275 |
| B/Ca | [2] | 3.266 | - | - | 211 | 189 | 238 |
| B/Ca | [2] | 3.317 | - | - | 254 | 225 | 278 |
| B/Ca | [2] | 3.322 | - | - | 267 | 240 | 295 |
| B/Ca | [2] | 3.327 | - | - | 229 | 206 | 258 |
| B/Ca | [2] | 3.338 | - | - | 247 | 219 | 271 |
| B/Ca | [2] | 3.343 | - | - | 266 | 239 | 294 |
| B/Ca | [2] | 3.348 | - | - | 237 | 211 | 263 |
| B/Ca | [2] | 3.354 | - | - | 243 | 216 | 270 |
| B/Ca | [2] | 3.363 | - | - | 279 | 251 | 308 |
| B/Ca | [2] | 3.368 | - | - | 271 | 242 | 297 |
| B/Ca | [2] | 3.373 | - | - | 289 | 260 | 317 |
| B/Ca | [2] | 3.383 | - | - | 312 | 285 | 345 |
| B/Ca | [2] | 3.388 | - | - | 302 | 277 | 336 |
| B/Ca | [2] | 3.393 | - | - | 308 | 282 | 341 |
| B/Ca | [2] | 3.396 | - | - | 277 | 252 | 308 |
| B/Ca | [2] | 3.401 | - | - | 267 | 242 | 298 |
| B/Ca | [2] | 3.406 | - | - | 261 | 237 | 292 |
| B/Ca | [2] | 3.41 | - | - | 255 | 229 | 283 |
| B/Ca | [2] | 3.415 | - | - | 245 | 220 | 272 |
| B/Ca | [2] | 3.42 | - | - | 274 | 248 | 304 |
| B/Ca | [2] | 3.447 | - | - | 266 | 241 | 295 |
| B/Ca | [2] | 3.606 | - | - | 270 | 243 | 299 |
| B/Ca | [2] | 3.658 | - | - | 256 | 231 | 286 |
| B/Ca | [2] | 3.78 | - | - | 192 | 168 | 215 |
| B/Ca | [2] | 3.959 | - | - | 216 | 191 | 242 |
| B/Ca | [2] | 4.057 | - | - | 234 | 210 | 263 |
| B/Ca | [2] | 4.14 | - | - | 206 | 181 | 229 |
| B/Ca | [2] | 4.142 | - | - | 209 | 185 | 234 |
| B/Ca | [2] | 4.146 | - | - | 234 | 209 | 262 |
| B/Ca | [2] | 4.15 | - | - | 185 | 163 | 211 |
| B/Ca | [2] | 4.154 | - | - | 247 | 220 | 274 |
| B/Ca | [2] | 4.158 | - | - | 263 | 239 | 295 |
| B/Ca | [2] | 4.162 | - | - | 269 | 245 | 302 |
| B/Ca | [2] | 4.166 | - | - | 236 | 211 | 265 |
| B/Ca | [2] | 4.171 | - | - | 215 | 190 | 240 |
| B/Ca | [2] | 4.173 | - | - | 223 | 198 | 250 |
| B/Ca | [2] | 4.177 | - | - | 209 | 186 | 236 |
| B/Ca | [2] | 4.181 | - | - | 224 | 201 | 254 |
| B/Ca | [2] | 4.185 | - | - | 241 | 218 | 273 |
| B/Ca | [2] | 4.189 | - | - | 223 | 199 | 251 |
| B/Ca | [2] | 4.193 | - | - | 207 | 183 | 232 |
| B/Ca | [2] | 4.197 | - | - | 215 | 188 | 239 |
| B/Ca | [2] | 4.2 | - | - | 235 | 212 | 265 |
| B/Ca | [2] | 4.2 | - | - | 225 | 202 | 254 |
| B/Ca | [2] | 4.21 | - | - | 216 | 192 | 243 |
| B/Ca | [2] | 4.21 | - | - | 226 | 201 | 254 |
| B/Ca | [2] | 4.22 | - | - | 216 | 191 | 242 |
| B/Ca | [2] | 4.22 | - | - | 244 | 219 | 273 |
| B/Ca | [2] | 4.22 | - | - | 249 | 224 | 278 |
| B/Ca | [2] | 4.23 | - | - | 212 | 188 | 237 |
| B/Ca | [2] | 4.23 | - | - | 204 | 181 | 229 |
| B/Ca | [2] | 4.23 | - | - | 214 | 192 | 242 |
| B/Ca | [2] | 4.24 | - | - | 219 | 195 | 245 |
| B/Ca | [2] | 4.24 | - | - | 186 | 163 | 209 |
| B/Ca | [2] | 4.25 | - | - | 221 | 196 | 247 |
| B/Ca | [2] | 4.25 | - | - | 194 | 171 | 218 |
| B/Ca | [2] | 4.25 | - | - | 203 | 180 | 229 |
| B/Ca | [2] | 4.26 | - | - | 226 | 202 | 254 |
| B/Ca | [2] | 4.26 | - | - | 210 | 185 | 234 |
| B/Ca | [2] | 4.27 | - | - | 220 | 196 | 246 |
| B/Ca | [2] | 4.27 | - | - | 219 | 195 | 246 |
| B/Ca | [2] | 4.27 | - | - | 191 | 169 | 216 |
| B/Ca | [2] | 4.28 | - | - | 230 | 204 | 256 |
| B/Ca | [2] | 4.28 | - | - | 230 | 206 | 258 |
| B/Ca | [2] | 5.06 | - | - | 331 | 305 | 371 |
| B/Ca | [2] | 5.08 | - | - | 309 | 283 | 347 |
| B/Ca | [2] | 5.16 | - | - | 237 | 212 | 270 |
| B/Ca | [2] | 7.27 | - | - | 308 | 282 | 351 |
| B/Ca | [2] | 7.68 | - | - | 216 | 186 | 242 |
| B/Ca | [2] | 8.09 | - | - | 221 | 191 | 249 |
| B/Ca | [2] | 8.5 | - | - | 236 | 220 | 255 |
| B/Ca | [2] | 8.91 | - | - | 242 | 212 | 276 |
| B/Ca | [2] | 9.33 | - | - | 240 | 208 | 273 |
| B/Ca | [2] | 9.74 | - | - | 247 | 217 | 284 |
| B/Ca | [2] | 12 | - | - | 368 | 324 | 439 |
| B/Ca | [2] | 13.06 | - | - | 405 | 362 | 476 |
| B/Ca | [2] | 15.04 | - | - | 450 | 407 | 496 |
| B/Ca | [2] | 15.99 | - | - | 407 | 366 | 454 |
| B/Ca | [2] | 17.16 | - | - | 408 | 367 | 455 |
| B/Ca | [2] | 18.1 | - | - | 426 | 384 | 474 |
| B/Ca | [2] | 19.06 | - | - | 386 | 346 | 435 |
| Paleosols | [3] | 12.8 | - | - | 433 | 305 | 561 |
| Paleosols | [3] | 13.1 | - | - | 519 | 262 | 776 |
| Paleosols | [3] | 13.8 | - | - | 310 | 233 | 387 |
| Paleosols | [3] | 14.4 | - | - | 203 | 133 | 273 |
| Paleosols | [3] | 14.7 | - | - | 116 | 79 | 153 |
| Paleosols | [3] | 15.1 | - | - | 291 | 209 | 373 |
| Paleosols | [3] | 15.6 | - | - | 852 | 766 | 938 |
| Paleosols | [3] | 16.0 | - | - | 579 | 489 | 669 |
| Phytoplankton | [4,5] | 5.4 | - | - | 268 | 247 | 340 |
| Phytoplankton | [4.5] | 5.8 | - | - | 246 | 227 | 312 |
| Phytoplankton | [4.5] | 6.1 | - | - | 256 | 236 | 324 |
| Phytoplankton | [4.5] | 6.4 | - | - | 259 | 239 | 328 |
| Phytoplankton | [4.5] | 6.8 | - | - | 286 | 263 | 366 |
| Phytoplankton | [4.5] | 7.2 | - | - | 249 | 229 | 314 |
| Phytoplankton | [4.5] | 7.3 | - | - | 271 | 251 | 344 |
| Phytoplankton | [4.5] | 7.6 | - | - | 237 | 218 | 300 |
| Phytoplankton | [4.5] | 8.3 | - | - | 244 | 225 | 309 |
| Phytoplankton | [4.5] | 8.7 | - | - | 254 | 235 | 321 |
| Phytoplankton | [4.5] | 9.1 | - | - | 234 | 215 | 295 |
| Phytoplankton | [4.5] | 9.1 | - | - | 276 | 254 | 352 |
| Phytoplankton | [4.5] | 9.6 | - | - | 265 | 245 | 336 |
| Phytoplankton | [4.5] | 9.6 | - | - | 271 | 250 | 343 |
| Phytoplankton | [4.5] | 9.6 | - | - | 241 | 222 | 304 |
| Phytoplankton | [4.5] | 9.6 | - | - | 211 | 193 | 266 |
| Phytoplankton | [4.5] | 9.8 | - | - | 254 | 234 | 321 |
| Phytoplankton | [4.5] | 9.9 |  |  | 242 | 223 | 304 |
| Phytoplankton | [4.5] | 10.1 |  |  | 252 | 233 | 319 |
| Phytoplankton | [4.5] | 10.2 |  |  | 262 | 242 | 332 |
| Phytoplankton | [4.5] | 10.2 |  |  | 251 | 231 | 318 |
| Phytoplankton | [4.5] | 10.3 |  |  | 250 | 231 | 316 |
| Phytoplankton | [4.5] | 10.5 |  |  | 258 | 238 | 327 |
| Phytoplankton | [4.5] | 10.6 |  |  | 229 | 212 | 287 |
| Phytoplankton | [4.5] | 10.7 |  |  | 253 | 233 | 320 |
| Phytoplankton | [4.5] | 10.9 |  |  | 234 | 216 | 295 |
| Phytoplankton | [4.5] | 10.9 |  |  | 245 | 226 | 309 |
| Phytoplankton | [4.5] | 11.1 |  |  | 226 | 209 | 285 |
| Phytoplankton | [4.5] | 11.3 |  |  | 239 | 220 | 301 |
| Phytoplankton | [4.5] | 11.4 |  |  | 217 | 200 | 274 |
| Phytoplankton | [4.5] | 11.5 |  |  | 210 | 194 | 262 |
| Phytoplankton | [4.5] | 11.6 |  |  | 232 | 213 | 292 |
| Phytoplankton | [4.5] | 11.9 |  |  | 252 | 233 | 318 |
| Phytoplankton | [4.5] | 12.1 |  |  | 219 | 201 | 276 |
| Phytoplankton | [4.5] | 12.2 |  |  | 208 | 191 | 261 |
| Phytoplankton | [4.5] | 12.2 |  |  | 214 | 197 | 269 |
| Phytoplankton | [4.5] | 12.3 |  |  | 219 | 202 | 276 |
| Phytoplankton | [4.5] | 12.5 |  |  | 224 | 206 | 284 |
| Phytoplankton | [4.5] | 12.7 |  |  | 216 | 198 | 272 |
| Phytoplankton | [4.5] | 12.7 |  |  | 208 | 192 | 263 |
| Phytoplankton | [4.5] | 12.8 |  |  | 230 | 212 | 291 |
| Phytoplankton | [4.5] | 12.9 |  |  | 217 | 200 | 272 |
| Phytoplankton | [4.5] | 12.9 |  |  | 235 | 216 | 296 |
| Phytoplankton | [4.5] | 13.1 |  |  | 211 | 195 | 265 |
| Phytoplankton | [4.5] | 13.2 |  |  | 232 | 214 | 291 |
| Phytoplankton | [4.5] | 13.3 |  |  | 237 | 218 | 299 |
| Phytoplankton | [4.5] | 13.3 |  |  | 235 | 217 | 297 |
| Phytoplankton | [4.5] | 13.4 |  |  | 227 | 209 | 285 |
| Phytoplankton | [4.5] | 13.4 |  |  | 230 | 212 | 288 |
| Phytoplankton | [4.5] | 13.5 |  |  | 230 | 212 | 288 |
| Phytoplankton | [4.5] | 13.5 |  |  | 209 | 193 | 262 |
| Phytoplankton | [4.5] | 13.6 |  |  | 194 | 178 | 243 |
| Phytoplankton | [4.5] | 13.7 |  |  | 219 | 202 | 277 |
| Phytoplankton | [4.5] | 14.1 |  |  | 237 | 219 | 300 |
| Phytoplankton | [4.5] | 14.2 |  |  | 226 | 208 | 286 |
| Phytoplankton | [4.5] | 14.5 |  |  | 211 | 194 | 265 |
| Phytoplankton | [4.5] | 14.9 |  |  | 243 | 223 | 307 |
| Phytoplankton | [4.5] | 15.0 |  |  | 236 | 218 | 297 |
| Phytoplankton | [4.5] | 15.1 |  |  | 226 | 208 | 285 |
| Phytoplankton | [4.5] | 15.1 |  |  | 221 | 204 | 280 |
| Phytoplankton | [4.5] | 15.2 |  |  | 193 | 177 | 242 |
| Phytoplankton | [4.5] | 15.3 |  |  | 205 | 189 | 257 |
| Phytoplankton | [4.5] | 15.4 |  |  | 208 | 192 | 260 |
| Phytoplankton | [4.5] | 15.5 |  |  | 211 | 195 | 265 |
| Phytoplankton | [4.5] | 16.1 |  |  | 219 | 202 | 275 |
| Phytoplankton | [4.5] | 16.2 |  |  | 181 | 167 | 227 |
| Phytoplankton | [4.5] | 16.3 |  |  | 184 | 170 | 230 |
| Phytoplankton | [4.5] | 16.3 |  |  | 245 | 226 | 310 |
| Phytoplankton | [4.5] | 16.4 |  |  | 206 | 190 | 260 |
| Phytoplankton | [4.5] | 16.6 |  |  | 226 | 208 | 286 |
| Phytoplankton | [4.5] | 16.8 |  |  | 240 | 233 | 327 |
| Phytoplankton | [4.5] | 17.3 |  |  | 239 | 220 | 301 |
| Phytoplankton | [4.5] | 17.3 |  |  | 213 | 208 | 289 |
| Phytoplankton | [4.5] | 17.3 |  |  | 211 | 205 | 287 |
| Phytoplankton | [4.5] | 17.4 |  |  | 229 | 223 | 313 |
| Phytoplankton | [4.5] | 17.5 |  |  | 222 | 216 | 302 |
| Phytoplankton | [4.5] | 17.5 |  |  | 236 | 229 | 322 |
| Phytoplankton | [4.5] | 17.5 |  |  | 244 | 238 | 332 |
| Phytoplankton | [4.5] | 17.6 |  |  | 228 | 210 | 289 |
| Phytoplankton | [4.5] | 17.6 |  |  | 223 | 217 | 304 |
| Phytoplankton | [4.5] | 17.6 |  |  | 218 | 213 | 297 |
| Phytoplankton | [4.5] | 17.7 |  |  | 224 | 219 | 305 |
| Phytoplankton | [4.5] | 17.7 |  |  | 224 | 218 | 304 |
| Phytoplankton | [4.5] | 17.8 |  |  | 227 | 221 | 307 |
| Phytoplankton | [4.5] | 17.8 |  |  | 240 | 234 | 326 |
| Phytoplankton | [4.5] | 17.8 |  |  | 226 | 221 | 308 |
| Phytoplankton | [4.5] | 17.9 |  |  | 208 | 203 | 283 |
| Phytoplankton | [4.5] | 17.9 |  |  | 223 | 217 | 302 |
| Phytoplankton | [4.5] | 18.1 |  |  | 234 | 216 | 296 |
| Phytoplankton | [4.5] | 18.3 |  |  | 259 | 239 | 327 |
| Phytoplankton | [4.5] | 18.3 |  |  | 227 | 222 | 307 |
| Phytoplankton | [4.5] | 18.4 |  |  | 227 | 222 | 309 |
| Phytoplankton | [4.5] | 18.5 |  |  | 228 | 222 | 309 |
| Phytoplankton | [4.5] | 18.5 |  |  | 221 | 204 | 279 |
| Phytoplankton | [4.5] | 18.6 |  |  | 239 | 220 | 302 |
| Phytoplankton | [4.5] | 18.6 |  |  | 223 | 205 | 280 |
| Phytoplankton | [4.5] | 18.9 |  |  | 231 | 225 | 314 |
| Phytoplankton | [4.5] | 19.0 |  |  | 236 | 218 | 299 |
| Phytoplankton | [4.5] | 19.2 |  |  | 224 | 206 | 283 |
| Phytoplankton | [4.5] | 19.2 |  |  | 243 | 237 | 331 |
| Phytoplankton | [4.5] | 19.4 |  |  | 236 | 230 | 320 |
| Phytoplankton | [4.5] | 19.5 |  |  | 220 | 215 | 299 |
| Phytoplankton | [4.5] | 19.7 |  |  | 217 | 212 | 296 |
| Phytoplankton | [4.5] | 20.0 |  |  | 265 | 258 | 363 |
| Phytoplankton | [4.5] | 20.0 |  |  | 255 | 235 | 324 |
| Phytoplankton | [4.5] | 20.2 |  |  | 214 | 197 | 270 |
| Phytoplankton | [4.5] | 20.3 |  |  | 313 | 302 | 430 |
| Phytoplankton | [4.5] | 20.4 |  |  | 367 | 353 | 503 |
| Phytoplankton | [4.5] | 20.5 |  |  | 329 | 317 | 453 |
| Phytoplankton | [4.5] | 20.5 |  |  | 214 | 196 | 270 |
| Phytoplankton | [4.5] | 20.8 |  |  | 289 | 280 | 393 |
| Phytoplankton | [4.5] | 20.8 |  |  | 211 | 194 | 267 |
| Phytoplankton | [4.5] | 20.8 |  |  | 208 | 191 | 261 |
| Phytoplankton | [4.5] | 20.8 |  |  | 321 | 310 | 440 |
| Phytoplankton | [4.5] | 20.9 |  |  | 247 | 227 | 313 |
| Phytoplankton | [4.5] | 21.1 |  |  | 269 | 247 | 344 |
| Phytoplankton | [4.5] | 21.5 |  |  | 295 | 286 | 404 |
| Phytoplankton | [4.5] | 22.2 |  |  | 326 | 314 | 447 |
| Phytoplankton | [4.5] | 22.7 |  |  | 266 | 258 | 363 |
| Phytoplankton | [4.5] | 23.3 |  |  | 217 | 199 | 274 |
| Phytoplankton | [4.5] | 23.8 |  |  | 264 | 243 | 337 |
| Phytoplankton | [4.5] | 23.9 |  |  | 258 | 250 | 353 |
| Phytoplankton | [4.5] | 23.9 |  |  | 336 | 324 | 464 |
| Phytoplankton | [4.5] | 23.9 |  |  | 278 | 257 | 353 |
| Phytoplankton | [4.5] | 24.2 |  |  | 264 | 244 | 333 |
| Phytoplankton | [4.5] | 24.4 |  |  | 253 | 233 | 323 |
| Phytoplankton | [4.5] | 24.5 |  |  | 229 | 210 | 290 |
| Phytoplankton | [4.5] | 24.5 |  |  | 306 | 295 | 421 |
| Phytoplankton | [4.5] | 24.6 |  |  | 264 | 256 | 362 |
| Phytoplankton | [4.5] | 24.7 |  |  | 423 | 404 | 586 |
| Phytoplankton | [4.5] | 24.7 |  |  | 473 | 413 | 660 |
| Phytoplankton | [4.5] | 24.7 |  |  | 750 | 634 | 1085 |
| Phytoplankton | [4.5] | 24.9 |  |  | 341 | 314 | 440 |
| Phytoplankton | [4.5] | 25.0 |  |  | 353 | 326 | 456 |
| Phytoplankton | [4.5] | 25.0 |  |  | 302 | 278 | 388 |
| Phytoplankton | [4.5] | 25.1 |  |  | 592 | 510 | 838 |
| Phytoplankton | [4.5] | 25.2 |  |  | 356 | 316 | 488 |
| Phytoplankton | [1] | 0.2 | - | - | 289 | 263 | 315 |
| Phytoplankton | [1] | 0.3 | - | - | 246 | 224 | 268 |
| Phytoplankton | [1] | 0.5 | - | - | 255 | 232 | 278 |
| Phytoplankton | [1] | 0.9 | - | - | 243 | 221 | 264 |
| Phytoplankton | [1] | 1.0 | - | - | 252 | 230 | 275 |
| Phytoplankton | [1] | 1.2 | - | - | 266 | 243 | 290 |
| Phytoplankton | [1] | 1.3 | - | - | 271 | 246 | 295 |
| Phytoplankton | [1] | 1.5 | - | - | 277 | 252 | 302 |
| Phytoplankton | [1] | 1.8 | - | - | 270 | 246 | 295 |
| Phytoplankton | [1] | 2.1 | - | - | 254 | 231 | 277 |
| Phytoplankton | [1] | 2.3 | - | - | 273 | 249 | 298 |
| Phytoplankton | [1] | 2.6 | - | - | 267 | 243 | 291 |
| Phytoplankton | [1] | 2.8 | - | - | 299 | 272 | 326 |
| Phytoplankton | [1] | 2.8 | - | - | 303 | 276 | 331 |
| Phytoplankton | [1] | 2.9 | - | - | 323 | 294 | 352 |
| Phytoplankton | [1] | 2.9 | - | - | 333 | 303 | 363 |
| Phytoplankton | [1] | 3.0 | - | - | 372 | 338 | 405 |
| Phytoplankton | [1] | 3.1 | - | - | 353 | 321 | 385 |
| Phytoplankton | [1] | 3.1 | - | - | 344 | 313 | 375 |
| Phytoplankton | [1] | 3.2 | - | - | 335 | 305 | 365 |
| Phytoplankton | [1] | 3.2 | - | - | 341 | 310 | 372 |
| Phytoplankton | [1] | 3.6 | - | - | 362 | 329 | 395 |
| Phytoplankton | [1] | 3.8 | - | - | 357 | 325 | 389 |
| Phytoplankton | [1] | 3.9 | - | - | 338 | 307 | 368 |
| Phytoplankton | [1] | 4.0 | - | - | 329 | 300 | 359 |
| Phytoplankton | [1] | 4.1 | - | - | 354 | 322 | 385 |
| Phytoplankton | [1] | 4.3 | - | - | 381 | 348 | 414 |
| Phytoplankton | [1] | 4.4 | - | - | 351 | 320 | 382 |
| Phytoplankton | [1] | 4.6 | - | - | 370 | 337 | 402 |
| Phytoplankton | [1] | 4.8 | - | - | 351 | 320 | 382 |
| Phytoplankton | [1] | 4.9 | - | - | 383 | 350 | 417 |
| Phytoplankton | [1] | 4.9 | - | - | 422 | 385 | 459 |
| Phytoplankton | [1] | 5.0 | - | - | 448 | 409 | 488 |
| Phytoplankton | [1] | 5.2 | - | - | 457 | 417 | 497 |
| Stomata | [6] | 11.6 | 11.0 | 12.2 | 332 | 315 | 348 |
| Stomata | [6] | 13.9 | 13.5 | 14.3 | 293 | 279 | 300 |
| Stomata | [6] | 15.7 | 15.0 | 16.4 | 469 | 410 | 449 |
| Stomata | [6] | 13.9 | 13.5 | 14.3 | 287 | 251 | 311 |
| Stomata | [6] | 15.7 | 15.0 | 16.4 | 555 | 371 | 788 |
| Stomata | [6] | 15.7 | 15.0 | 16.4 | 552 | 357 | 787 |
| Stomata | [6] | 15.8 | 15.6 | 16.0 | 564 | 350 | 793 |
| Stomata | [7] | 3.89 | 3.6 | 4.2 | 355 | 306 | 811 |
| Stomata | [7] | 0.01 | 0.0117 | 0.0139 | 270 | 233 | 287 |
| Stomata | [7] | 3.9 | 3.6 | 4.2 | 355 | 306 | 811 |
| Stomata | [7] | 12.0 | 11.6 | 12.3 | 468 | 382 | 661 |
| Stomata | [8,9] | 15.2 | 13.7 | 16.7 | 338 | 304 | 705 |
| Stomata | [8,9] | 15.3 | 13.8 | 16.8 | 338 | 304 | 705 |
| Stomata | [8,9] | 15.3 | 13.8 | 16.8 | 314 | 304 | 591 |
| Stomata | [10,11] | 1.95 | 1.8 | 2.1 | 358 | 340 | 375 |
| Stomata | [10,11] | 4 | - | - | 363 | 345 | 380 |
| Stomata | [10,11] | 2.7 | - | - | 276 | 260 | 292 |
| Stomata | [10,11] | 3.4 | - | - | 358 | 340 | 375 |
| Stomata | [12] | 2.65 | 2.52 | 2.77 | 534 | 493 | 574 |
| Stomata | [13] | 3.05 | 2.7 | 3.4 | 351 | 333 | 378 |

1. Seki O, Foster GL, Schmidt DN, Mackensen A, Kawamura K, et al. (2010) Alkenone and boron-based Pliocene pCO_2_ records. Earth and Planetary Science Letters 292: 201-211.

2. Tripati AK, Roberts CD, Eagle RA (2009) Coupling of CO_2_ and ice sheet stability over major climate transitions of the last 20 million years. Science 326: 1394-1397.

3. Retallack GJ (2009) Refining a pedogenic-carbonate CO_2_ paleobarometer to quantify a middle Miocene greenhouse spike. Palaeogeography Palaeoclimatology Palaeoecology 281: 57-65.

4. Henderiks J, Pagani M (2008) Coccolithophore cell size and the Paleogene decline in atmospheric CO_2_. Earth and Planetary Science Letters 269 576-584.

5. Pagani M, Lemarchand D, Spivack A, Gaillardet J (2005) A critical evaluation of the boron isotope-pH proxy: The accuracy of ancient ocean pH estimates. Geochimica Et Cosmochimica Acta 69: 953-961.

6. Kürschner WM, Kvacek Z, Dilcher DL (2008) The impact of Miocene atmospheric carbon dioxide fluctuations on climate and the evolution of terrestrial ecosystems. Proc Natl Acad Sci U S A 105: 449-453.

7. Retallack GJ (2009) Greenhouse crises of the past 300 million years. Geological Society of America Bulletin 121: 1441--1455.

8. Beerling DJ, Fox A, Anderson CW (2010) Quantitative uncertainty analyses of ancient atmospheric CO2 estimates from fossil leaves. American Journal of Science 309: 775-787.

9. Royer DL (2001) Stomatal density and stomatal index as indicators of paleoatmospheric CO_2_ concentration. Review of palaeobotany and palynology 114: 1-28.

10. Van Der Burgh J, Visscher H, Dilcher DL, Kurschner WM (1993) Paleoatmospheric signatures in neogene fossil leaves. Science 260: 1788-1790.

11. Kürschner WM, van der Burgh J, Visscher H, Dilcher DL (1996) Oak leaves as biosensors of late neogene and early pleistocene paleoatmospheric CO_2_ concentrations. Marine Micropaleontology 27: 299-312.

12. Bai YJ, Chen LQ, Ranhotra PS, Wang Q, Wang YF, et al. (2014) Reconstructing atmospheric CO_2_ during the Plio-Pleistocene transition by fossil Typha. Glob Chang Biol.

13. Stults DZ, Wagner-Cremer F, Axsmith BJ (2011) Atmospheric paleo-CO_2_ estimates based on Taxodium distichum (Cupressaceae) fossils from the Miocene and Pliocene of Eastern North America. Palaeogeography Palaeoclimatology Palaeoecology 309: 327-332.
